# Novel PCR Primers for the Archaeal Phylum *Thaumarchaeota* Designed Based on the Comparative Analysis of 16S rRNA Gene Sequences

**DOI:** 10.1371/journal.pone.0096197

**Published:** 2014-05-07

**Authors:** Jin-Kyung Hong, Hye-Jin Kim, Jae-Chang Cho

**Affiliations:** Institute of Environmental Sciences and Department of Environmental Sciences, Hankuk University of Foreign Studies, Yong-In, Korea; Graz University of Technology (TU Graz), Austria

## Abstract

Based on comparative phylogenetic analysis of 16S rRNA gene sequences deposited in an RDP database, we constructed a local database of thaumarchaeotal 16S rRNA gene sequences and developed a novel PCR primer specific for the archaeal phylum *Thaumarchaeota*. Among 9,727 quality-filtered (chimeral-checked, size >1.2 kb) archaeal sequences downloaded from the RDP database, 1,549 thaumarchaeotal sequences were identified and included in our local database. In our study, *Thaumarchaeota* included archaeal groups MG-I, SAGMCG-I, SCG, FSCG, RC, and HWCG-III, forming a monophyletic group in the phylogenetic tree. Cluster analysis revealed 114 phylotypes for *Thaumarchaeota*. The majority of the phylotypes (66.7%) belonged to the MG-I and SCG, which together contained most (93.9%) of the thaumarchaeotal sequences in our local database. A phylum-directed primer was designed from a consensus sequence of the phylotype sequences, and the primer’s specificity was evaluated for coverage and tolerance both *in silico* and empirically. The phylum-directed primer, designated THAUM-494, showed >90% coverage for *Thaumarchaeota* and <1% tolerance to non-target taxa, indicating high specificity. To validate this result experimentally, PCRs were performed with THAUM-494 in combination with a universal archaeal primer (ARC917R or 1017FAR) and DNAs from five environmental samples to construct clone libraries. THAUM-494 showed a satisfactory specificity in empirical studies, as expected from the *in silico* results. Phylogenetic analysis of 859 cloned sequences obtained from 10 clone libraries revealed that >95% of the amplified sequences belonged to *Thaumarchaeota*. The most frequently sampled thaumarchaeotal subgroups in our samples were SCG, MG-I, and SAGMCG-I. To our knowledge, THAUM-494 is the first phylum-level primer for *Thaumarchaeota*. Furthermore, the high coverage and low tolerance of THAUM-494 will make it a potentially valuable tool in understanding the phylogenetic diversity and ecological niche of *Thaumarchaeota*.

## Introduction

The archaeal phylum *Thaumarchaeota* was proposed in 2008, distinguishing mesophilic ammonia-oxidizing archaeal (AOA) lineages from hyperthermophilic *Crenarchaeota* lineages [1]. This proposal was based on archaeal phylogeny inferred from rRNA and ribosomal protein sequences, which suggested that mesophilic *Crenarchaeota* constitute a distinct phylum that branches off near the root of *Archaea*. A few years later, this distinction was confirmed by genomic information (e.g., the identification of *Thaumarchaeota*-specific genes) in *Cenarchaeum symbiosum*, *Nitrosopumilus maritimus*, and *Nitrososphaera gargensis*, representatives of marine and terrestrial AOA lineages [2].

The discovery of *Thaumarchaeota* sparked interest not only in the field of microbial ecology, but also in the fields of evolution, physiology, and molecular biology of the domain *Archaea*, since the majority of its members described so far are mesophilic ammonia oxidizers [3]. In the past, the *Archaea* were thought to be confined to extreme environments [4]; consequently, their ecological role in global geochemical cycling was underestimated. However, a number of molecular ecological studies have revealed that the *Archaea* inhabit a wide variety of moderate environments [5], suggesting that they play a substantial role in global geochemical cycling. Based on 16S rRNA gene surveys, the *Thaumarchaeota* have been estimated to represent up to 20% and 5% of all prokaryotes in marine and terrestrial environments, respectively [6]–[8]. Another notable feature of the *Thaumarchaeota* is that all cultured or enriched members of this phylum are ammonia oxidizers [9]–[16]. Before AOA were discovered, ammonia oxidation was thought to be performed exclusively by ammonia-oxidizing bacteria (AOB) in the bacterial phylum *Proteobacteria* [17]. Initial evidence supporting archaeal ammonia oxidation included the discovery of archaeal homologs of bacterial ammonia monooxigenase genes (*amoA* and *amoB*) in metagenomes [18], [19]. Later, additional studies concluded that *amoA*-carrying archaea are AOA, and suggested that AOA could contribute significantly to the global nitrogen cycle [9], [10], [12], [20]–[23]. Recent studies have also shown that the copy numbers of archaeal *amoA* are much higher than the copy numbers of bacterial *amoA* in many soil samples [24]–[29], indicating the predominance of AOA over AOB.

Since AOA are highly fastidious organisms (only a few laboratories have successfully isolated or enriched AOA from the environment), most ecological studies of AOA depend on PCR-based molecular methods. Hence, PCR primer specificity inevitably affects analysis and interpretation. However, PCR primers specific for AOA or *Thaumarchaeota* have not been well established. Moreover, all 16S rRNA primers previously used to quantify AOA targeted a single thaumarchaeotal subgroup [28], [30]–[37]. Almost all molecular ecological studies of AOA or the *Thaumarchaeota* assumed that marine group 1.1a AOA (hereafter referred to as MG-I) and soil group 1.1b AOA (hereafter referred to as SCG) predominated in marine and terrestrial samples, respectively. Thus, most of these studies employed primers specific to one of these subgroups for estimating the abundance of AOA or *Thaumarchaeota*. Although different thaumarchaeotal subgroups have been hypothesized to have different niches [28], [38], [39], samples could potentially harbor an unexpected AOA subgroup (e.g., subgroup MG-I in soil samples, or subgroup SCG in marine samples), as observed by Tourna et al. [37] and Beman and Francis [40]; moreover, samples could also harbor multiple subgroups or even as-yet-undiscovered subgroups. In such samples, the abundance and diversity of AOA or *Thaumarchaeota* could be drastically underestimated. We attributed the lack of phylum-level primers (*Thaumarchaeota*-directed primers) to the ambiguously defined phylogenetic range of *Thaumarchaeota* and the limited number of thaumarchaeotal 16S rRNA sequences available at the time of primer design. However, the current availability of a large sequence database has facilitated the timely design of PCR primers covering the entire phylogenetic range of *Thaumarchaeota*. Such primers will contribute to our understanding of the ecological roles, distribution patterns, and environmental factors shaping the niche of this phylum.

In this study, we constructed a local database of thaumarchaeotal 16S rRNA gene sequences by comparatively analyzing all 16S rRNA gene sequences deposited in an RDP database, and developed a phylum-directed primer for *Thaumarchaeota*. The specificity of the designed primer was assessed by comparing its performance to those of existing subgroup-directed primers. To the best of our knowledge, this is the first study to comprehensively analyze the thaumarchaeotal 16S rRNA gene sequences with the most current database, and to design a *Thaumarchaeota*-directed primer. Herein, we describe the phylogenetic diversity and breadth of the phylum *Thaumarchaeota* and the specificity of the newly designed PCR primer.

## Materials and Methods

### Construction of Local Database

**Figure 1:**
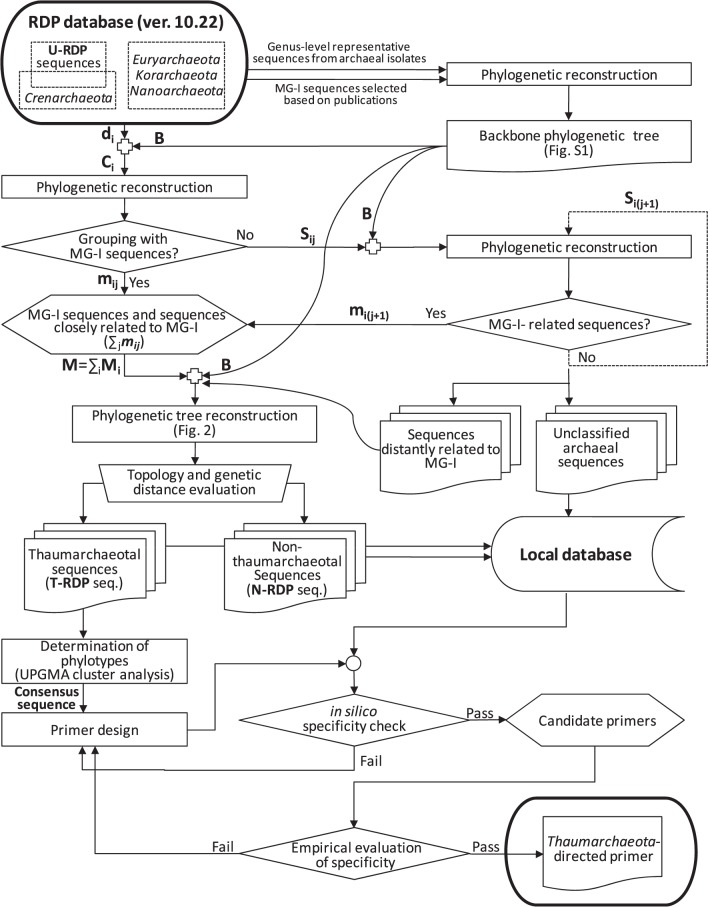
Schematic diagram showing the overall process to construct local database and to design primers. Bold letters indicate sequence sets (or subsets). Open cross symbols and dashed lines indicate sequence merge points and repeating sub-routines, respectively.

Thaumarchaeotal 16S rRNA gene sequences were collected from the RDP database (release 10.22, 2010, http://rdp.cme.msu.edu) [41]. Because no sequences were found under the database category, “phylum *Thaumarchaeota*,” in the RDP database, we first identified *Thaumarchaeota*-related sequences throughout the RDP database using an iterative phylogenetic approach (Fig. 1).

Using the selection criteria employed by the RDP website, chimera check (pass) and length (near full-length [>1.2 kb]) of sequences, a total of 9,727 sequences out of 62,077 archaeal sequences were downloaded from the RDP database to construct our local database (Table 1). Prior to the iteration routine for searching and collecting the *Thaumarchaeota*-related sequences from the downloaded sequences, a backbone phylogenetic tree (Fig. S1) was constructed with representative sequences of archaeal taxa (see below for the phylogenetic reconstruction). The sequence set for the backbone tree, B  =  {backbone sequences}, included 106 sequences from the group MG-I as key members of the phylum *Thaumarchaeota*, which were selected based on published literature [12], [42]–[49], and 72 genus-level representative sequences (type strains and genome-sequenced strains) from the phyla *Euryarchaeota*, *Crenarchaeota*, *Nanoarchaeota*, and *Korarchaeota* (Table. S1).

**Table 1:** Summary of thaumarchaeotal 16S rRNA gene sequences used for phylogenetic analysis and primer design.

Taxa^a^	RDP database^b^		Local database	
	No. of total sequences	No. of quality-filtered^c^ sequences	No. of sequences	Mean size (base) ± SD^d^
*Crenarchaeota*	13,316	2,616	872	1,373.7±89.0
*Thermoprotei*	13,316 (11,406)^e^	2,616 (1,837)^e^	872 (93)^e^	1,376.3±88.5
*Euryarchaeota*	35,985	6,072	6,072	1,366.6±80.7
*Archaeoglobi*	415	70	70	1,408.8±65.7
*Halobacteria*	4,576	1,377	1,377	1,411.6±60.9
*Methanobacteria*	5,222	728	728	1,302.9±71.5
*Methanococci*	474	126	126	1,398.4±65.6
*Methanomicrobia*	14,270	2,058	2,058	1,377.9±59.8
*Methanopyri*	18	5	5	1,400.6±100.2
*Thermococci*	670	229	229	1,426.4±94.5
*Thermoplasmata*	1,615	437	437	1,326.2±74.8
Unclassified *Euryarchaeota*	8,725	1,042	1,042	1,326.1±90.7
*Korarchaeota*	213	88	88	1,306.0±75.2
*Nanoarchaeota*	54	3	3	1,473.3±23.1
*Thaumarchaeota*	-f	-	1,549	1,376.1±54.3
FSCG^g^	-	-	17	1,384.1±56.1
HWCG-III^g^	-	-	25	1,342.4±49.3
MG-I^g^	-	-	1,100	1,373.1±56.4
RC^g^	-	-	7	1,372.1±57.9
SAGMCG-I^g^	-	-	40	1,359.8±43.4
SCG^g^	-	-	355	1,389.5±45.1
UT-I^h^	-	-	2	1,405.5±6.4
UT-II^h^	-	-	1	1,232^k^
UT-III^h^	-	-	2	1,333.0±5.7
DSAG^i^	-	-	326	1,293.9±77.2
THSCG^i^	-	-	50	1,340.6±44.3
MCG^i^	-	-	483	1,342.8±91.0
UG^i^	-	-	12	1,345.4±84.9
Unclassified *Archaea* j	12,509	948	272	1,330.3±75.0
Total	62,077	9,727	9,727	1,363.4±79.8

a Phylum- and class-level archaeal groups.

b RDP database release 10.22.

c Filtered using quality check option (http://rdp.cme.msu.edu).

d Standard deviation.

e Sequences belong to unclassified *Thermoprotei.*

f Not shown in RDP database.

g Subgroups in *Thaumarchaeota* (sequences closely related to MG-I). FSCG (forest soil crenarchaeotic group); HWCG-III (hot water crenarchaeotic group III); MG-I (marine group I); RC (rice cluster); SAGMCG-I (South Africa gold mine crenarchaeotic group I); SCG (soil crenarchaeotic group).

h Unclassified *Thaumarchaeota*. Unclassified thaumarchaeotal subgroups found in this study.

i Archaeal groups distantly related to the phylum *Thaumarchaeota*. DSAG (deep sea archaeotic group); THSCG (terrestrial hot spring crenarchaeotic group); MCG (miscellaneous crenarchaeotic group); UG (unclassified group).

j As shown in RDP database.

k Standard deviation not available.

In the first round of the iteration routine (*i* = 1 and *j* = 1), a subset of downloaded RDP sequences, d*_i_*, was added to the sequence set B, resulting in a combined sequence set, C*_i_* = B+d*_i_*. Then a subset of MG-I-related sequences, m*_ij_*, in the sequence set d*_i_* (m*_ij_*⊂d*_i_*), was identified from a phylogenetic tree constructed with the sequence set C*_i_* (see below for the phylogenetic reconstruction). The input sequences that formed a tight (bootstrap score >80%) monophyletic cluster with the MG-I sequences that were included in the sequence set B were regarded as ‘MG-I-related sequences’. In the following rounds (*j* = *j*+1), MG-I-related sequences were searched repeatedly in a phylogenetic tree constructed with a subtracted sequence set, S*_i_*_(*j*+1)_ = S*_ij_*−m*_ij_* (S*_ij_* = C*_i_*−m*_ij_*, if *j* = 1) until no more MG-I-related sequences were found in the sequence set S*_i_*_(*j*+1)_. After collecting MG-I-related sequences (M*_i_* = ∑*_j_* m*_ij_*) from the sequence subset d*_i_*, the monophyly of the sequence set M*_i_* was evaluated again with the bootstrap score, then the iteration routine was performed for the remaining subsets of the RDP sequences, d*_i_*_+1_. Finally, the downloaded RDP sequences that formed a monophyletic cluster (∑*_i_*M*_i_*) were regarded as thaumarchaeotal sequences (T) in our local database. Our local database sequences are available at http://sdrv.ms/1k8frAc with RDP’s structure-based alignment format.

The final version of phylogenetic tree was constructed with the thaumarchaeotal sequences (T) identified during the iteration routine. Backbone sequences (B) were included in the phylogenetic tree to show the phylogenetic position of the phylum *Thaumarchaeota*. Some sequences distantly related to the group MG-I were also included in the final version of the phylogenetic tree for later reference.

### Estimating Genetic Distances and Rarefaction Analysis

Pair-wise genetic distances between sequences in the local database were measured with MEGA [50] using the Jukes-Cantor (JC) model [51] and were subjected to UPGMA (unweighted pair group method with arithmetic mean) cluster analysis implemented in MEGA. Thaumarchaeotal phylotypes were defined by a cophenetic distance of 0.2 (sequence similarity ≥98%) in the cluster analysis, which roughly corresponded to species-level 16S rRNA gene similarity [52]. Intra- and inter-subgroup genetic distances were estimated from the phylotype sequences of the archaeal groups listed in Table 1. For the phyla *Crenarchaeota*, *Euryarchaeota*, *Korarchaeota*, and *Nanoarchaeota*, only the genus-level representative sequences included in the backbone sequence set (B) were used for the estimation of the genetic distances due to the calculation load.

Rarefaction analysis was performed to estimate the phylotype richness of the phylum *Thaumarchaeota*. Phylotypes were defined as operational taxonomic units (OTUs), and the OTU richness estimators (*S_obs_*) for the phylum *Thaumarchaeota* and each of its subgroups were calculated using EstimateS [53].

### Phylogenetic Reconstruction

A multiple alignment of the phylotype sequences determined by the cluster analysis was subjected to phylogenetic reconstruction. A bacterial sequence (GenBank accession no. J01695) was used as an outgroup, and sequences belonging to archaeal phyla other than the phylum *Thaumarchaeota* were also included in the phylogenetic analysis. Phylogenetic trees were inferred using the neighbor joining (NJ) algorithm implemented in the MEGA [50]. The tree topology was statistically evaluated by 100 bootstrap re-samplings and was further confirmed using the maximum likelihood (ML) algorithm (GTR+CAT approximation) implemented in the RAxML [54].

### Local Bayesian Classifier

A local naïve Bayesian classifier was built with our local database to serve as a training set of sequences. The algorithm previously established by Wang et al [55], which is currently implemented in RDP’s classifier, was used to estimate the probability that a query sequence, S, is a member of phylum D, *P*(D|S) = *P*(S|D)×*P*(D)/*P*(S), where *P*(D) is the prior probability of a sequence being a member of phylum D and *P*(S) is the overall probability of finding sequence S in any phyla. The joint probability of observing the sequence S, which contains a set of words (subsequences, v_i_), was estimated as *P*(S|D) = ∏ *P*(v_i_|D). Word-specific priors were calculated with the 8-base subsequences, and the priors *P*(D) and *P*(S) were assumed to be constant terms according to the original paper [55]. The query sequences that gave the highest probability were classified as being members of the phylum D.

### Primer Design

A phylum-directed primer for the *Thaumarchaeota* was designed with a thaumarchaeotal consensus sequence using Primrose [56]. The consensus sequence (majority rule) for the phylum *Thaumarchaeota* was obtained from the thaumarchaeotal phylotypes in the local database. We permitted no degenerate site in the primer sequences. The specificity of the designed primer (or primer pairs used) was evaluated using Oligocheck (www.cf.ac.uk/biosi/research/biosoft/) with local database sequences and determined both by the extent to which the primer binds to target group sequences (coverage) and non-target sequences (tolerance). The tolerance of the primer to domain *Bacteria* was evaluated using the ProbeMatch program implemented in the RDP website. The thermodynamic properties (e.g., free energy, *ΔG*, predictions) for self-complementary structures (hair-pin and primer-dimer) were determined using NetPrimer (http://www.premierbiosoft.com/netprimer/).

### PCR Amplification

Soil samples were collected from Kellerberrin in Western Australia, Hillgate in southern California, La Campana in central Chile and Incheon in Korea, and a marine sediment sample was collected from the continental shelf of the Yellow Sea, west of Jeju Island, Korea. Details of the sampling locations and the physicochemical properties of the samples were described elsewhere [57], [58]. Community DNAs were directly extracted from the samples using the MoBio PowerSoil DNA extraction kit (MoBio Laboratories, Solana Beach, Calif., USA) according to the manufacturer’s protocol and were individually used as PCR templates.

The phylum-directed primer designed in this study (primer THAUM-494) was used with the universal primer ARC917R or 1017FAR [59], [60]. The reaction mixture included 25 µl of TaKaRa Ex Taq premix (Takara, Shiga, Japan), 1 µl of each forward and reverse primer (stock concentration, 20 µM), 200 ng of template DNA extracted from the soil sample, and sterilized distilled water to give a 50 µl final volume. The PCR thermal profile was as follows: initial denaturation at 95°C for 5 min, followed by 30 cycles consisting of denaturation at 95°C for 30 s, primer annealing at 55°C for 30 s, and extension at 72°C for 30 s. The final elongation step was extended to 20 min. PCR amplification was performed with a GeneAmp PCR system 9700 (Applied Biosystems, Foster City, Calif., USA). Positive PCR amplicons were confirmed using agarose gel electrophoresis.

### Cloning

The PCR amplicons were purified using a QIAquick PCR purification kit (Qiagen, Valencia, Calif., USA) and cloned into TOPO cloning vectors with a TOPO TA cloning kit (Invitrogen, Carlsbad, Calif., USA) to construct the clone libraries according to the manufacturer’s protocol. Insert sequences of 859 clones, which were randomly selected from 10 clone libraries (ca. 90 clones per primer pairs used and 180 clones per sample) were sequenced using an ABI3700 DNA analyzer (Applied Biosystems, Foster City, Calif., USA). The phylogenetic affiliations of the cloned sequences were determined using phylogenetic reconstruction and were further confirmed by our local Bayesian classifier. When the local Bayesian classifier applied, the cloned sequences were classified as being members of the phylum D or its subgroup O that gave the highest probability, *P*(D|S) or *P*(O|S), where S was a cloned sequence as a query.

### Nucleotide Sequence Accession Numbers

All sequences produced in this study have been deposited in GenBank under the accession numbers KF275675 to KF276604.

## Results and Discussion

### Overview of Thaumarchaeotal Sequences in Public Databases

To design taxon-directed primers (or probes), the entire taxonomic range of the target taxon should be clearly defined so that designers can easily distinguish sequences belonging to non-target taxa from those belonging to the target taxon. While prokaryote taxonomy should not be solely deduced from 16S rRNA gene sequences, *a priori* knowledge of the phylogenetic breadth of the target taxon, which is partially reflected in the 16S rRNA-based phylogenetic tree, is a prerequisite for developing primers that specifically bind to complementary regions of target 16S rRNA gene sequences. However, our knowledge regarding the phylogenetic range of *Thaumarchaeota* is rather limited, with the exceptions of certain subgroups (MG-I, SCG, and HWCG-III [hot water crenarchaeotic group III]) used to define this phylum [1]. A large number of archaeal 16S rRNA gene sequences have been deposited into public databases (e.g., 128,378 and 38,641 in RDP [release 10.32, 2013] and SILVA [release 114, 2013] databases, respectively). However, only two studies, both of which comprehensively analyzed archaeal 16S rRNA sequences that are either closely or distantly related to *Thaumarchaeota*, have attempted to define the phylogenetic range of *Thaumarchaeota* [61], [62]. These two studies both classified the archaeal groups MG-I, SCG, SAGMCG-I (South Africa gold mine crenarchaeotic group I), and HWCG-III within the phylum *Thaumarchaeota*. However, these two studies reached contradictory conclusions regarding classification of the other subgroups.

The SILVA database (release 114, 2013, http://arb-silva.de) [63] assigns 15,773 16S rRNA gene sequences to the phylum *Thaumarchaeota*, and divides *Thaumarchaeota* into 27 subgroups. However, this classification is mainly based on the phylogenetic assignment of the 16S rRNA gene sequences according to literature, with the phylogenetic positions of those sequences determined by SILVA’s workflow (personal communication). To date, no supporting materials have been published regarding SILVA’s classification scheme for thaumarchaeotal 16S rRNA gene sequences. For example, the SILVA database classifies 16S rRNA gene sequences of MCG (miscellaneous crenarchaeotic group, previously named TMCG, where “T” stands for “terrestrial”), DSAG (deep sea archaeal group, alternatively named MBG-B, marine benthic group B), and HWCG-I (hot water crenarchaeotic group I) into *Thaumarchaeota*. However, recent studies have concluded that rRNA-based phylogenies are insufficient for determining the relationship of these three archaeal groups to *Thaumarchaeota*, proposing that more data are required to accurately define their taxonomic positions [62], [64]. The situation is more complicated with other 16S rRNA gene-oriented databases. For example, the RDP database does not retrieve any 16S rRNA gene sequences when the *Thaumarchaeota* taxonomic categories used. Moreover, the Greengenes database (2013 version, http://greengenes.lbl.gov) [65] does not even index the phylum *Thaumarchaeota*. Due to the lack of robust phylogenetic affiliations and the difficulties in accessing *Thaumarchaeota*-related sequences in current public databases, we constructed our own local database of thaumarchaeotal 16S rRNA gene sequences to facilitate the development of *Thaumarchaeota*-directed primers.

### Collection of *Thaumarchaeota*-related Sequences

We initially constructed a backbone phylogenetic tree (Fig. S1) for the domain *Archaea*. This tree was built with 106 MG-I sequences, selected from the published literature, and 72 representative sequences from well-defined taxa in the archaeal phyla *Crenarchaeota*, *Euryarchaeota*, *Korarchaeota*, and *Nanoarchaeota* (Table. S1). Since MG-I is a basal constituent of the phylum *Thaumarchaeota* [1], the only thaumarchaeotal sequences initially included in the backbone phylogenetic tree were these 106 MG-I sequences. *Thaumarchaeota* sequences were limited in this way to avoid phylogenetic inferences for other thaumarchaeotal subgroups at the initial stages of sequence collection, as well as to define the minimum phylogenetic range of *Thaumarchaeota*. In this backbone tree, the MG-I sequences formed a tight monophyletic cluster (bootstrap score, 100%), comprising a lineage distinct from *Crenarchaeota*, *Euryarchaeota*, *Korarchaeota*, and *Nanoarchaeota*. While *Crenarchaeota* was a monophyletic group (bootstrap score, 98%), *Nanoarchaeota* and *Korarchaeota* were not-well-resolved in this tree. In addition, *Euryarchaeota* was split into four independent clusters and appeared to be paraphyletic, as previously reported [66], [67]. However, further attempts to clarify the phylogenetic positions of these unresolved phyla were left for future studies, since phylogenetic inference for archaeal phyla other than *Thaumarchaeota* was considered beyond the scope of this study. In subsequent iteration steps for collecting thaumarchaeotal sequences (Fig. 1), 16S rRNA gene sequences belonging to archaeal phyla other than *Thaumarchaeota* were considered to be non-target (non-thaumarchaeotal) sequences. All sequences forming a tight monophyletic cluster (bootstrap score >80%) with MG-I sequences were collected and classified as thaumarchaeotal.

**Figure 2:**
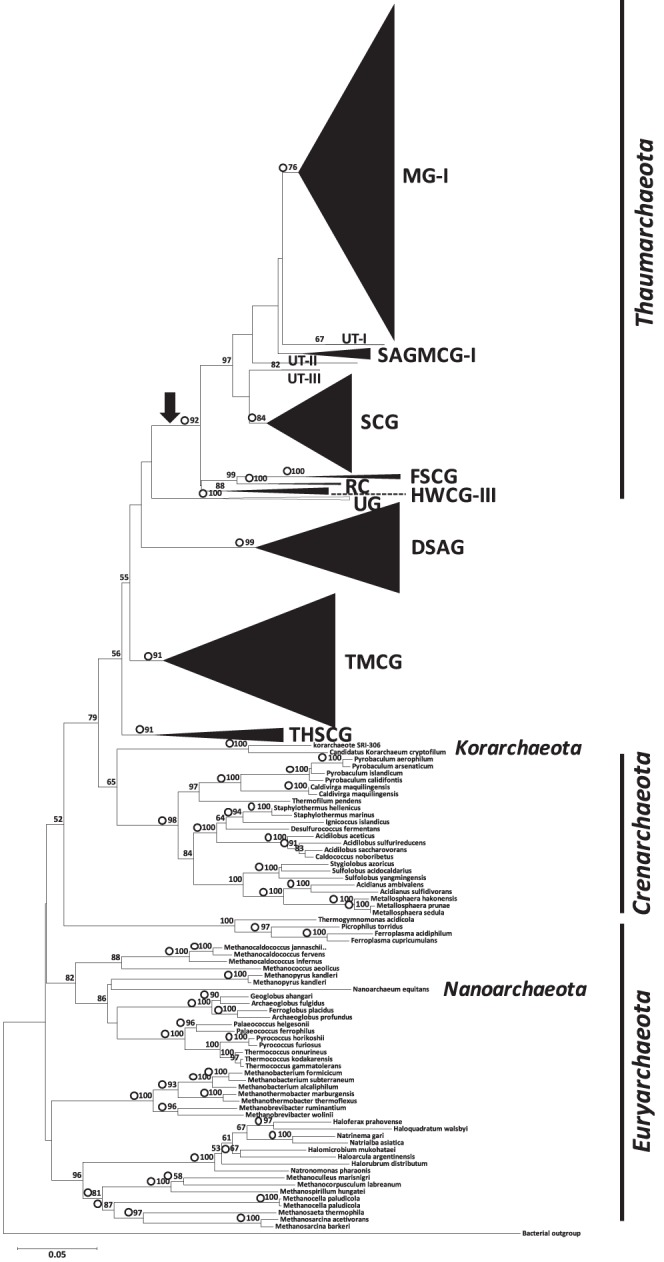
Phylogenetic positions of archaeal sequences in our local database. The phylogenetic distances of each sequence were calculated using the Jukes-Cantor model, and the tree was constructed using the neighbor-joining (NJ) algorithm. The numbers at the nodes indicates the bootstrap score (as a percentage) and are shown for the frequencies at or above the threshold of 50%. Open circles indicate the bootstrap score >50% estimated using the randomized accelerated maximum likelihood (RAxML) algorithm (GTR+CAT approximation). Arrow indicates an internal node corresponding to the phylum *Thaumarchaeota*. The scale bar represents the expected number of substitutions per nucleotide position.

During iteration routine performed with the 178 sequences included in the backbone tree and the 9,727 RDP quality-filtered archaeal sequences downloaded from the RDP database, 272 RDP sequences could not be assigned to any archaeal group in the backbone tree due to their unstable phylogenetic positions near the root of the domain *Archaea*. These sequences were assigned to ‘unclassified *Archaea*’ in our local database. With the exceptions of the RDP sequences that had been properly classified as *Euryarchaeota*, *Nanoarchaeota*, or *Korarchaeota* at the phylum-level in the RDP database (n = 6,163), we found that 2,419 RDP sequences (hereafter referred to as U-RDP sequences) formed clusters distinct from *Crenarchaeota, Euryarchaeota*, *Nanoarchaeota*, and *Korarchaeota.* All of these U-RDP sequences were derived from RDP taxa designated as either ‘unclassified *Thermoprotei*’ in the phylum *Crenarchaeota* (n = 1,596) or ‘unclassified *Archaea*’ (n = 823) (Table 1). A neighbor joining (NJ) phylogenetic tree was constructed with these U-RDP sequences and backbone sequences (Fig. 2); in this tree, 1,549 U-RDP sequences (hereafter referred to as T-RDP sequences) formed a tight monophyletic cluster with MG-I sequences, which was supported by a high bootstrap score (92%). The monophyly of the T-RDP cluster was also confirmed in a maximum likelihood (ML) tree, with an associated bootstrap score of 95%. We assigned this monophyletic group to the phylum *Thaumarchaeota*, containing nine subgroups in this study (Table 1; Table S2). Among these subgroups, six corresponded to previously recognized archaeal groups: MG-I, SAGMCG-I, SCG, FSCG (forest soil crenarchaeotic group), RC (rice cluster), and HWCG-III. The remaining subgroups, comprised of five sequences (AB050231, AB113628, EF444677, HM187528, and HM187541), were assigned to groups designated as UT-1, -2, and -3 (UT, unclassified *Thaumarchaeota*) in our local database, since their previous designations were unclear or unavailable. Two UT-1 sequences, AB050231 and AB113628, had previously been reported as SAGMCG-II sequences in previous studies [48], [68], whereas the other U-RDP sequences belonging to SAGMCG-II were merged into the MG-I cluster in our phylogenetic tree. Among the six subgroups corresponding to known archaeal groups, five subgroups (MG-I, SCG, FSCG, RC, and HWCG-III) appeared to be monophyletic (bootstrap scores >50%).

The remaining U-RDP sequences (hereafter referred to as N-RDP sequences, n = 870) were clustered into four independent groups, whose cohesive phylogenetic relationships to known archaeal phyla were not supported by high bootstrap scores (bootstrap scores <50%) in either NJ or ML trees. Three groups of these N-RDP sequences corresponded to previously reported archaeal groups: DSAG, MCG, and THSCG (terrestrial hot spring crenarchaeotic group), whereas the remaining N-RDP group was arbitrarily designated as 'unclassified group’ (UG) in this study. These UG sequences had also been designated as unclassified archaea in previous studies [42], [45], [48], [69], [70]. In addition to phylogenetic tree topologies, the inter-group genetic distances between MG-I and the four N-RDP sequence groups (0.283±0.028) were as great as those between the recognized archaeal phyla *Crenarchaeota*, *Euryarchaeota*, *Nanoarchaeota*, and *Korarchaeota* (inter-phylum genetic distance, 0.297±0.023) (Table 2). On the other hand, inter-group genetic distances between MG-I and its close sister groups (FSCG, HWCG-III, RC, SAGMCG-I, SCG, and UTs) (0.165±0.035) were significantly smaller (α = 0.05, *t*-test) than the average inter-phylum genetic distance, thus supporting our initial hypothesis that these groups belong to the phylum *Thaumarchaeota*. Consequently, we classified MG-I, FSCG, HWCG-III, RC, SAGMCG-I, SCG, and UTs into *Thaumarchaeota* in our local database and considered DSAG, MCG, THSCG, and UG to be distinct phylum-level groups, not affiliated with any recognized archaeal phylum. Consistent with our results, Pester et al. [62] showed that *Thaumarchaeota* included the archaeal groups MG-I, SCG, HWCG-III, SAGMCG-I, and FSCG. Pester and colleagues also noted that DSAG and MCG are not clearly affiliated with any established archaeal phylum. In the final version of our local database, we noticed that sequences belonging to another recently proposed archaeal phylum, *Aigarchaeota* [71], were included in the phylum *Crenarchaeota*. No close relationships between aigarchaeotal sequences and T-RDP sequences were observed during our analysis.

**Table 2:** Genetic distances between archaeal taxa (lower left half, mean genetic distance; upper right half, standard deviation) included in the local database^a^.

Taxa	Cren	Eury	Kor	Nano	DSAG	MCG	THSCG	UG	Thaum	FSCG	HWCG-III	MG-I	RC	SAGMCG-I	SCG	UT-I	UT-II	UT-III
*Crenarchaeota* (Cren)		0.073	0.056	0.062	0.046	0.065	0.065	0.063	0.057	0.062	0.058	0.051	0.059	0.053	0.063	0.058	0.051	0.054
*Euryarchaeota* (Eury)	0.302		0.051	0.064	0.038	0.047	0.052	0.041	0.030	0.032	0.041	0.024	0.034	0.033	0.034	0.031	0.029	0.032
*Korarchaeota* (Kor)	0.260	0.307		0.018	0.016	0.023	0.024	0.027	0.024	0.030	0.023	0.074	0.059	0.064	0.076	0.077	0.099	0.075
*Nanoarchaeota* (Nano)	0.277	0.318	0.316		0.013	0.013	0.014	0.024	0.024	0.029	0.014	0.014	0.006	0.011	0.010	0.007	NA^b^	0.003
DSAG	0.341	0.347	0.362	0.415		0.023	0.012	0.014	0.016	0.022	0.016	0.016	0.017	0.016	0.014	0.013	0.012	0.017
MCG	**0.246** c	0.296	0.268	0.323	0.304		0.020	0.020	0.027	0.019	0.020	0.014	0.033	0.015	0.012	0.017	0.012	0.017
THSCG	**0.232**	0.296	**0.248**	0.312	0.317	**0.233**		0.017	0.027	0.024	0.019	0.017	0.034	0.020	0.017	0.019	0.018	0.019
Unclassified group (UG)	0.275	0.312	0.292	0.351	0.316	**0.219**	0.261		0.025	0.020	0.018	0.022	0.033	0.014	0.017	0.015	0.023	0.014
*Thaumarchaeota* (Thaum)	0.308	0.342	0.304	0.391	0.321	0.275	0.254	0.283										
FSCG	0.288	0.332	0.309	0.367	0.338	0.255	**0.234**	0.287			0.027	0.026	0.034	0.025	0.028	0.026	0.026	0.026
HWCG-III	**0.239**	0.296	**0.245**	0.326	0.305	**0.225**	**0.198**	**0.246**		**0.182**		0.015	0.010	0.014	0.016	0.015	0.016	0.015
MG-I	0.321	0.348	0.316	0.402	0.323	0.288	0.268	0.292		**0.234**	**0.193**		0.010	0.015	0.013	0.010	0.009	0.010
RC	0.279	0.319	0.287	0.375	0.338	**0.238**	**0.228**	0.264		**0.174**	**0.176**	**0.199**		0.077	0.067	0.068	0.073	0.071
SAGMCG-I	0.294	0.334	0.285	0.376	0.323	0.262	**0.242**	0.274		**0.219**	**0.171**	**0.123**	**0.166**		0.015	0.014	0.008	0.021
SCG	0.274	0.327	0.273	0.364	0.315	**0.243**	**0.217**	0.260		**0.200**	**0.136**	**0.174**	**0.175**	**0.155**		0.020	0.012	0.022
UT-I	0.291	0.335	0.283	0.377	0.317	0.264	**0.240**	0.270		**0.225**	**0.166**	**0.124**	**0.191**	**0.135**	**0.118**		0.106	0.094
UT-II	0.307	0.353	0.300	0.387	0.346	0.275	0.253	0.296		**0.229**	**0.172**	**0.146**	**0.196**	**0.157**	**0.116**	**0.170**		0.008
UT-III	0.270	0.316	0.276	0.350	0.323	**0.249**	**0.236**	0.267		**0.200**	**0.152**	**0.146**	**0.160**	**0.095**	**0.108**	**0.127**	**0.132**	

a For the phyla *Crenarchaeota, Euryarchaeota, Korarchaeota*, and *Nanoarchaeota*, the backbone sequences were used to estimate the genetic distances.

b Not available due to the insufficient number of comparisons.

c Genetic distances less than 0.25 are in bold.

**Figure 3:**
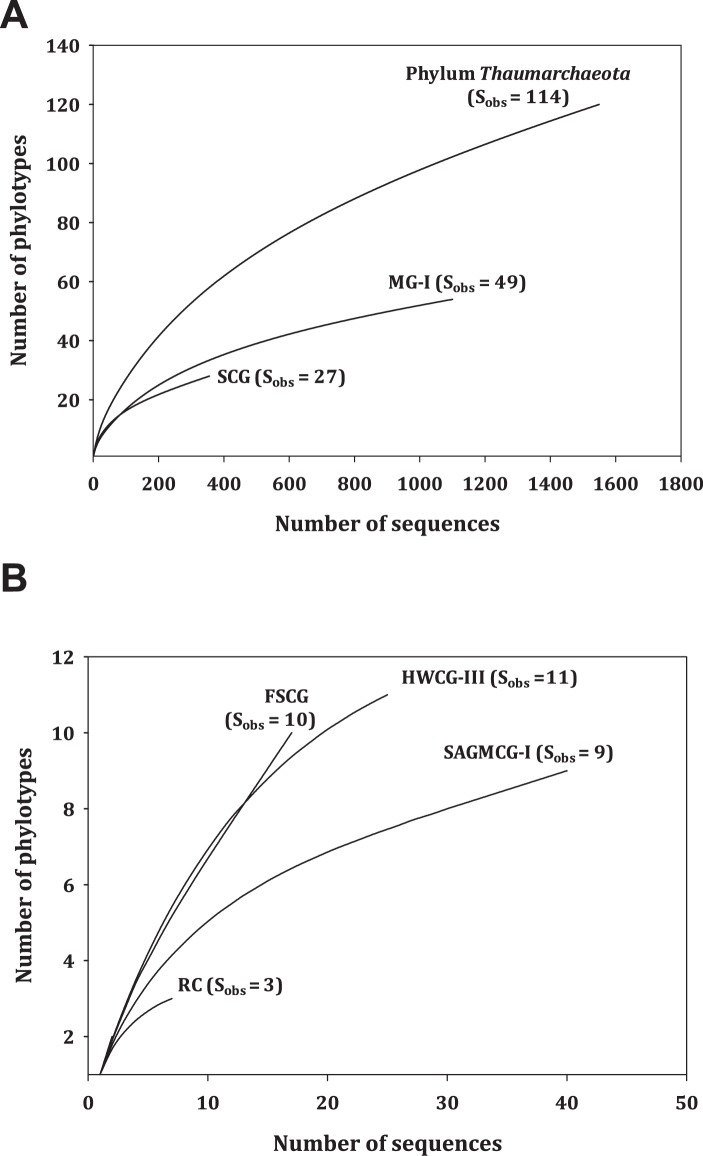
Rarefaction curves for the phylum *Thaumarchaeota* and its subgroups. Phylotypes were defined as operational taxonomic unit (OTU). (A) Phylum *Thaumarchaeota* and subgroups MG-I and SCG. (B) Subgroups FSCG, HWCG-III, RC, and SAGMCG-I.

Using cluster analysis (cutoff level, 98% cophenetic similarity), thaumarchaeotal phylotypes were defined with 1,549 thaumarchaeotal 16S rRNA gene sequences (T-RDP sequences; average size, 1376.7±54.3 bases) in the local database. In total, 114 phylotypes were observed (Table 3), with the majority (66.7%) belonging to the MG-I and SCG subgroups, which together contained most (93.9%) of the thaumarchaeotal sequences in our local database. No plateaus were observed on *Thaumarchaeota* rarefaction curves (Fig. 3), suggesting that, on a global scale, sequence sampling is still inadequate for accurately estimating the extent of diversity within the phylum *Thaumarchaeota*. However, the numbers of sequences per phylotype within the MG-I and SCG subgroups were much larger than those for other subgroups (Table 3), indicating that the majority of the sequences currently appended to these two groups actually belong to previously sampled phylotypes.

**Table 3:** Number of phylotypes and intra-group genetic distances of archaeal groups included in the local database.

Taxa	Intra-group genetic distance^a^ (mean ± SD^b^)	No. of phylotype	Average no. of sequences per phylotype
*Crenarchaeota*	0.152±0.050	-c	-
*Euryarchaeota*	0.253±0.075	-	-
*Korarchaeota*	0.089^d^	-	-
*Nanoarchaeota*	NA^e^	-	-
DSAG	0.086±0.059	-	-
MCG	0.146±0.043	-	-
THSCG	0.091±0.060	-	-
UG	0.124±0.036	-	-
*Thaumarchaeota*	0.153±0.054	114	14.3
FSCG	0.108±0.028	10	1.7
HWCG-III	0.097±0.027	11	2.3
MG-I	0.083±0.022	49	25.0
RC	0.082±0.033	3	2.3
SAGMCG-I	0.074±0.022	9	4.3
SCG	0.078±0.021	27	12.5
UT-I	0.074^d^	2	1.0
UT-II	NA	1	1.0
UT-III	0.048^d^	2	1.0

a Intra-group genetic distances for *Crenarchaeota, Euryarchaeota, Korarchaeota*, and *Nanoarchaeota* were estimated from the backbone sequences.

b Standard deviation.

c Not determined.

d Standard deviation not available.

e Not available due to the insufficient number of sequences.

### Design and *In silico* Evaluation of the *Thaumarchaeota*-directed Primer

Taking into consideration the specificity for its intended target sequences, as well as its thermodynamic propensities to form self-complementary structures (such as hair-pins and primer-dimers), we developed a *Thaumarchaeota*-directed primer, henceforth referred to as THAUM-494, from the consensus sequence of all thaumarchaeotal phylotype sequences (Table 3; Table S3). The specificity of THAUM-494, defined in terms of its coverage and tolerance, was evaluated *in silico* with our local database. We defined primer coverage as the extent to which the primer binds its target group sequences, tolerance as the extent to which the primer binds non-target sequences; both of these parameters for THAUM-494 were compared with those of previously designed primers (or probes) targeting sequences belonging to MG-I or mesophilic *Crenarchaeota* (Table 4 and 5; Table S4). THAUM-494 showed >90% coverage for *Thaumarchaeota* and <1% tolerance to non-target taxa, indicating high specificity for the phylum *Thaumarchaeota*. All non-target sequences (n = 8) with regions complementary to THAUM-494 belonged to the class *Thermoprotei* of the phylum *Crenarchaeota*. Further examination of the non-target sequences binding THAUM-494, using our local Bayesian classifier, revealed that they were all highly related to the thaumarchaeotal subgroups SCG or HWCG-III. THAUM-494 covered the major subgroups MG-I, SCG, and SAGMCG-I at a rate of >90% each. THAUM-494 did not bind to sequences in FSCG and RC, two minor subgroups comprising only 1.5% of all thaumarchaeotal sequences.

**Table 4:** Primers used for PCR and *in silico* evaluation of the specificity.

Primer	Target taxon	Sequence (5′→3′)	Sequence position		%GC	No.of degenerate sites	Thermodynamic properties^a^				Reference
			*E. coli*	*M. jannasch*			Rating	Tm	Hairpin ΔG	Dimer ΔG	
THAUM-494	Phylum*Thaumarchaeota*	GAATAAGGGGTGGGCAAGT	494–511	435–453	52.6	0	100	56.4	0.0	0.0	This study
CREN512	Phylum*Crenarchaeota*	CTGGTGTCAGCCGCCG	512–527	454–469	75.0	0	100	59.1	0.0	0.0	Jürgens et al., 2000
542F	Phylum*Crenarchaeota*	CGCGGTAATACCAGCYC	526–542	468–484	62.5	1	81	52.2	0.0	–10.4	Hershberger et al., 1996
Cren745a	Phylum*Crenarchaeota*	GGTGAGGGATGAAAGCTGGG	755–774	696–717	60.0	0	86	61.6	0.0	–7.3	Simon et al., 2000
Cren518R	Phylum*Crenarchaeota*	TCAGCCGCCGCGGTAAWACCAGC	518–541	460–482	68.2	1	68	74.7	–1.5	–16.5	Perevalova et al., 2003
GI–554	Group MG-I	AGGAKGATTATTGGGCCTAA	554–573	496–515	42.1	1	79	51.6	–1.2	–10.3	Massana et al., 1997
GI_751F	Group MG-I	GTCTACCAGAACAYGTTC	734–751	672–692	47.1	1	89	36.2	0.0	–5.9	Gubry-Rangin et al., 2010
771F	Group MG-I	ACGGTGAGGGATGAAAGCT	753–771	694–712	52.6	0	86	56.7	0.0	–7.3	Ochsenreiter et al., 2003
GI_956R	Group MG-I	CAATTGGAGTCAACGCCD	957–974	903–920	52.9	0	83	51.6	0.0	–9.3	Beman et al., 2008
957R	Group MG-I	CAATTGGAGTCAACGCCG	957–974	903–920	55.6	0	83	57.7	0.0	9.3	Ochsenreiter et al., 2003
MCGI-554r^b^	Group MG-I	CAGCACCTCAAGTGGTCA	537–554	479–496	55.6	0	88	51.8	–1.1	–5.4	Auguet et al., 2012
MCGI-391f	Group MG-I	AAGGTTARTCCGAGTGRTTTC	391–422	376–396	42.1	2	100	48.8	0.0	0.0	Auguet et al., 2012
333Fa^b^	Domain*Archaea*	TCCAGGCCCTACGGG	334–348	320–333	73.3	0	80	54.3	–1.9	–9.3	Baker et al., 2003
Arch338F	Domain*Archaea*	GGCCCTAYGGGGYGCASCAGGC	338–359	324–344	79.0	3	63	68.3	–4.8	–16.4	Kublanov et al., 2009
340RA	Domain*Archaea*	CCRGGCCCTACGGGG	335–349	321–334	85.7	1	77	57.6	–3.4	–9.8	Barns et al., 1994
A340F	Domain*Archaea*	CCCTACGGGGYGCASCAG	340–357	326–342	75.0	2	97	56.9	–1.7	0.0	Vetriani et al., 1999
ARC349F	Domain*Archaea*	GYGCASCAGKCGMGAAW	349–365	334–350	66.7	5	100	39.4	0.0	0.0	Takai and Horikoshi, 2000
EK510R	Domain*Archaea*	AAGGGCYGGGCAAG	498–510	439–452	69.2	1	100	48.9	0.0	0.0	Baker et al., 2003
ARC516	Domain*Archaea*	TGYCAGCCGCCGCGGTAAHACCVGC	516–541	458–482	72.7	3	55	78.9	–10.8	–16.5	Takai and Horikoshi, 2000
PARCH519F	Domain*Archaea*	CAGCMGCCGCGGTAA	519–533	461–475	71.4	1	68	55.2	0.0	–17.5	Øvreås et al., 1997
A751F	Domain*Archaea*	CCGACGGTGAGRGRYGAA	750–767	691–708	66.7	3	100	51.8	0.0	0.0	Baker et al., 2003
744RA	Domain*Archaea*	GGATTAGATACCCSGG	785–800	726–741	53.3	1	80	40.9	0.0	–10.8	Barns et al., 1994
ARC806R	Domain*Archaea*	ATTAGATACCCSBGTAGTCC	787–806	728–747	44.4	2	100	43.0	0.0	0.0	Takai and Horikoshi, 2000
765FA	Domain*Archaea*	TAGATACCCSSGTAGTCC	789–806	730–747	50.0	2	100	37.7	0.0	0.0	Barns et al., 1994
Ar9R	Domain*Archaea*	GAAACTTAAAGGAATTGGCGGG	906–927	851–872	45.5	0	90	62.2	0.0	–5.4	Jürgens et al., 2000
ARC915R^b^	Domain*Archaea*	AGGAATTGGCGGGGGAGCAC	915–934	860–879	65.0	0	90	67.8	0.0	–5.4	Casamayor et al., 2000
ARC917R	Domain*Archaea*	GAATTGGCGGGGGAGCAC	915–934	860–879	66.7	0	90	63.2	0.0	–5.4	Loy et al., 2002
Arch958R^b^	Domain*Archaea*	AATTGGAKTCAACGCCGGR	958–975	904–921	52.9	2	80	56.9	0.0	–10.8	DeLong, 1992
1017FAR^b^	Domain*Archaea*	GAGAGGWGGTGCATGGCC	1044–1060	982–999	70.6	1	81	58.6	0.0	–10.3	Barns et al., 1994
D34	Domain*Archaea*	CAGGCAACGAGCGAGACC	1096–1113	1035–1052	66.7	0	100	59.5	0.0	0.0	Arahal et al., 1996
A1098F	Domain*Archaea*	GGCAACGAGCGMGACCC	1098–1114	1037–1053	75.0	1	100	59.7	0.0	0.0	Baker et al., 2003
A1115R	Domain*Archaea*	CAACGAGCGAGACCC	1100–1114	1039–1053	66.7	0	100	48.5	0.0	0.0	Baker et al., 2003

a Calculated using NetPrimer (http://www.premierbiosoft.com/netprimer). T_m_ was estimated using the Nearest neighbor method implemented in the NetPrimer.

b Primers MCGI-554r, 333Fa, ARC915R, Arch958R, and 1017FAR are identical to CREN537, A333F, A934R, A976R, and A1040F, respectively.

**Table 5:** *In silico* evaluation (percent matched 16S rRNA gene sequences in the target taxon) of the specificity of *Thaumarchaeota-* and *Crenarchaeota-*directed primers.

Taxa	No. of sequences used for evaluation	*Thaumarchaeota-*directed primer	*Crenarchaeota-*directed primer/probe				MG-I-directed primer/probe						
		THAUM-494	542F	CREN512	Cren745a	Cren518R	GI554	771F	957R	GI751F	GI956R	MCGI391f (MGI391)	MCGI554r (Cren537)
*Crenarchaeota*	872	0.9	**86.4**	**90.0**	11.0	**86.5**		10.9	0.6		51.0		
*Euryarchaeota*	6,072		≈0		≈0				≈0		≈0		
*Korarchaeota*	88												
*Nanoarchaeota*	3												
*Thaumarchaeota*	1,549	**92.9** a	1.4	96.6	94.3	95.9	65.4	94.6	22.4	29.6	95.4	35.8	65.5
FSCG	17		82.4	88.2	76.5	82.4		76.5			94.1		
HWCG-III	25	36.0		76.0	100	96.0		100			76.0		
MG-I	1,100	**96.6**		97.3	94.8	95.7	**92.1**	**95.2**	0.1	41.8	**96.5**	50.4	**92.3**
RC	7		100	100	85.7	100		85.7			100		
SAGMCG-I	40	**100**		97.5	85.0	97.5		87.5	10.0		97.5		
SCG	355	**90.7**		96.3	94.1	96.6		94.1	96.1		93.0		
UT-I	2	**100**		100	100	100		100	50.0		100		
UT-II	1	**100**		100	100	100		100					
UT-III	2	**100**		100	100	100		100			100		
DSAG	326												
THSCG	50		92.0	94.0		92.0					2.0		
MCG	483		88.2	89.6	27.3	88.0	0.2	28.0	0.2		25.5		
UG	12			100									
Unclassified *Archaea*	272		41.9	44.9	41.2	42.3		41.2	0.4		41.3		
Domain *Bacteria* b	667,899												

a Coverage values of more than 80% for the target taxa are in bold, and tolerance values of more than 1% to the non-target taxa are under-lined.

b Estimation using RDP’s ProbeMatch.

In addition to THAUM-494, we also used our local database to evaluate the specificity of previously designed primers (probes) targeting *Thaumarchaeota*-related taxa (Table 5; Table S4). The first oligonucleotide sequence specifically designed to target one of the thaumarchaeotal subgroups was the probe GI-554 [72], developed for MG-I (crenarchaeal group I in original paper) in 1997. Although the number of available MG-I sequences was limited at the time, GI-554 showed satisfactory coverage (92.1%) for MG-I and extremely low (0.2%) non-target binding in our *in silico* analysis. However, as intended, GI-554 bound only to MG-I sequences and did not bind to sequences in other thaumarchaeotal subgroups. Several years later, three primer pairs, 771F-957R [7], [33], [35], [73], GI751F-GI956R [31], [74], [75], and MCGI391f-MCGI554r (identical to MGI391 and Cren537) [30], [36], [76], were developed to amplify the 16S rRNA gene sequences of terrestrial crenarchaeota, nitrifying archaea, and MG-I, respectively. Our *in silico* results showed that 771F had relatively high coverage (94.6%) for *Thaumarchaeota*, but also had high tolerance to *Crenarchaeota* (10.9%), as well as other non-target groups (>28.0%). The reverse partner of 771F, 957R, covered only SCG sequences (96.1%) and exhibited low tolerance (<1%) to non-thaumarchaeotal sequences. Similarly, GI-751F bound 41.8% of all MG-I sequences, but did not cover other thaumarchaeotal subgroups. GI-956R showed high coverage (95.4%) for *Thaumarchaeota*, but was also highly tolerant to *Crenarchaeota* (tolerance, 51.0%). Primer MCGI391f (MGI391) showed a specificity similar to the primer GI-751F, with slightly increased coverage (50.4%) for MG-I. The coverage and tolerance of MCGI554r (Cren537) were very similar to those of GI-554. Primers 542F, CREN512, Cren745a, and Cren518, originally designed to target the mesophilic group of *Crenarchaeota* when the current thaumarchaeotal subgroups (MG-I, SCG, and FSCG) were considered to belong to *Crenarchaeota* [77]–[80], had high coverage not only for *Crenarchaeota*, but also for *Thaumarchaeota*. In summary, the previously designed primers with high coverage for the phylum *Thaumarchaeota* showed high tolerance to non-thaumarchaeotal taxa, and the primers with low tolerance to non-thaumarchaeotal taxa showed low coverage for *Thaumarchaeota*.

### Empirical Evaluation of the *Thaumarchaeota*-directed Primer

To empirically evaluate the specificity of THAUM-494, we constructed clone libraries using PCR products obtained with THAUM-494 (Table 6). For a reverse primer, one of universal primers, ARC917R [60] or 1017FAR [59] was used, and environmental DNA extracted from soil and marine samples was used as a PCR template. The *in silico* coverage estimates of the universal primers ARC917R and 1017FAR for *Thaumarchaeota* were 96.1% and 82.2%, respectively (Table 7 and Table 8). Among the primer pair combinations (THAUM-494 and universal primer) that generated PCR amplicons >400 bp in size, primer pair THAUM-494-ARC917R demonstrated the highest coverage (89.3%) for *Thaumarchaeota* (Table 8). Five clone libraries were constructed for each primer pair. Phylogenetic positions of cloned sequences were determined from phylogenetic trees built with reference sequences (Fig. S2–S6) and were confirmed with our local Bayesian classifier, developed with the local database sequences.

**Table 6:** Empirical evaluation of the specificity (percentage of cloned sequences belonging to the phylum *Thaumarchaeota* and its subgroups) of primer THAUM-494.

Sample		Reverse primer	Phylum *Thaumarchaeota*	Thaumarchaeotal subgroups							No. of clones analyzed
Site	Type			MG-I	SCG	SAGMCG-I	HWCG-III	FSCG	RC	UTs^a^	
Hillgate, California	Woodland	ARC917R	96.3	1.2	95.1						81
		1017FAR	100		71.6					28.4	102
Incheon, Korea	Paddy soil	ARC917R	98.9	5.5		68.1				25.3	91
		1017FAR	98.9	3.3	1.1	71.1				23.3	90
Jeju, Korea	Marine sediment	ARC917R	90.9	87.9		1.5		1.5			66
		1017FAR	85.4	80.5	1.2					3.7	82
Kellerberrin, Austrailia	Woodland	ARC917R	92.3	11.0	80.2			1.1			91
		1017FAR	100		96.4					3.6	83
La Campana, Chile	Woodland	ARC917R	98.9	2.1	96.8						94
		1017FAR	100		97.5					2.5	79
Total		ARC917R	95.5±3.7^b^								423
		1017FAR	96.9±6.4								436

a UT-I, -II, and -III.

b Standard deviation.

**Table 7:** *In silico* evaluation (percent matched 16S rRNA gene sequences) of the specificity of primer pairs (THAUM-494-universal primer).

Taxa	No. of sequences used for evaluation	Universal primers									
		333Fa (A333F)	Arch338F	340RA	A340F	ARC349F	EK510R	ARC516	PARCH519F	A751F	744RA
*Crenarchaeota*	872	0 (8.4)^a^	0.6 (74.7)	0 (**85.7** b)	0 (**86.9**)	0.7 (71.9)	0 (0.1)	0.9 (**94.6**)	0.9 (**96.8**)	0.3 (**90.0**)	0.9 (49.8)
*Euryarchaeota*	6,072	0 (55.2)	0 (78.1)	0 (**81.9**)	0 (**91.2**)	0 (**86.6**)	0 (46.3)	0 (**81.1**)	0 (**96.8**)	0 (32.9)	0 (**93.0**)
*Korarchaeota*	88		0 (22.7)	0 (9.1)	0 (25.0)	0 (19.3)		0 (26.1)	0 (**93.2**)	0 (**92.0**)	0 (**96.6**)
*Nanoarchaeota*	3				0 (33.0)						
*Thaumarchaeota*	1,549	0 (0.1)	**85.9** b (**90.1**)	0.1 (0.3)	0.1 (0.1)	**91.0** (**97.2**)		**89.6** (**95.9**)	**91.1** (**97.4**)	35.6 (36.9)	**90.5** (**97.0**)
FSCG	17	0 (11.8)		0 (11.8)		0 (**82.4**)		0 (**82.4**)	0 (**82.4**)		0 (**82.4**)
HWCG-III	25					24.0 (56.0)		36.0 (**96.0**)	36.0 (**96.0**)	36.0 (36.0)	32.0 (**96.0**)
MG-I	1,100		**88.6** (**91.8**)			**94.7** (**98.0**)		**92.8** (**95.9**)	**94.7** (**97.8**)	49.4 (51.1)	**94.5** (**97.5**)
RC	7					0 (**100**)		0 (**100**)	0 (**100**)		0 (**100**)
SAGMCG-I	40		**95.0** (**95.0**)			**97.5** (**97.5**)		**97.5** (**97.5**)	**97.5** (**97.5**)		**95.0** (**95.0**)
SCG	355		**88.2** (**96.3**)	0.6 (0.6)	0.6 (0.6)	**89.3** (**98.0**)		**88.5** (**96.3**)	**89.0** (**96.9**)		**87.9** (**96.3**)
UT-I	2		**100** (**100**)			**100** (**100**)		**100** (**100**)	**100** (**100**)		**100** (**100**)
UT-II	1		**100** (**100**)			**100** (**100**)		**100** (**100**)	**100** (**100**)		**100** (**100**)
UT-III	2		**100** (**100**)			**100** (**100**)		**100** (**100**)	**100** (**100**)		**100** (**100**)
DSAG	326		0 (3.7)	0 (9.5)	0 (9.5)	0 (20.6)		0 (**95.1**)	0 (**96.6**)	0 (5.5)	0 (**92.9**)
THSCG	50	0 (74.0)	0 (**88.0**)	0 (**92.0**)	0 (**90.0**)	0 (**94.0**)		0 (**92.0**)	0 (**96.0**)	0 (**94.0**)	0 (**98.0**)
MCG	483	0 (20.9)	0 (11.8)	0 (**86.1**)	0 (**86.1**)	0 (68.5)		0 (**90.7**)	0 (**94.0**)	0 (37.5)	0 (**88.4**)
UG	12	0 (25.0)	0 (**100**)	0 (**83.3**)	0 (**100**)	0 (**100**)		0 (**83.3**)	0 (**91.7**)		0 (58.3)
Unclassified *Archaea*	272	0 (35.3)	0 (55.9)	0 (47.8)	0 (77.6)	0 (**86.0**)		0 (50.7)	0 (**86.8**)	0 (28.7)	0 (**89.0**)
Domain *Bacteria* c	667,899								(92.3 b)		

a Coverage values of universal primer.

b Coverage values of more than 80% for the target taxa are in bold, and tolerance values of more than 1% to the non-target taxa are under-lined.

c Estimation using RDP’s ProbeMatch.

**Table 8:** *In silico* evaluation (percent matched 16S rRNA gene sequences) of the specificity of primer pairs (THAUM-494-universal primer).

Taxa	No. of sequences used for evaluation	Universal primers									
		ARC806R	765FA	Ar9R	ARC915R	ARC917R	Arch958R	1017FAR (A1040F)	D34	A1098F	A1115R
*Crenarchaeota*	872	0.9 (**85.1** b)^a^	0.9 (53.7)	0.9 (**92.3**)	0.9 (75.5)	0.9 (76.3)	0.8 (51.9)	0.9 (74.8)	0.1 (26.3)	0.1 (61.6)	0.1 (61.8)
*Euryarchaeota*	6,072	0 (**91.5**)	0 (**91.8**)	0 (**91.5**)	0 (**91.2**)	0 (**91.8**)	0 (45.3)	0 (**84.0**)	0 (59.2)	0 (58.8)	0 (59.1)
*Korarchaeota*	88	0 (**93.2**)	0 (**93.2**)						0 (**92.0**)	0 (**92.0**)	0 (**92.0**)
*Nanoarchaeota*	3										
*Thaumarchaeota*	1,549	**89.5** (**95.9**)	**89.9** (**96.3**)	**89.2** (**95.7**)	**89.2** (**95.9**)	**89.3** (**96.1**)	20.3 (23.4)	76.0 (**82.2**)	0.3 (1.7)	0.3 (1.7)	0.3 (1.7)
FSCG	17	0 (**82.4**)	0 (**82.4**)	0 (**88.2**)	0 (**100**)	0 (**100**)		0 (**100**)	0 (**100**)	0 (**100**)	0 (**100**)
HWCG-III	25	28.0 (**88.0**)	28.0 (**88.0**)	32.0 (**96.0**)	32.0 (**92.0**)	32.0 (**92.0**)	36.0 (**100**)	36.0 (**100**)	12.0 (12.0)	12.0 (12.0)	12.0 (12.0)
MG-I	1,100	**93.3** (**96.3**)	**93.5** (**96.5**)	**93.4** (**96.5**)	**92.7** (**95.7**)	**92.9** (**96.0**)	0.4 (0.4)	73.5 (75.6)	0.2 (0.2)	0.2 (0.2)	0.2 (0.2)
RC	7	0 (**100**)	0 (**100**)	0 (**100**)	0 (**100**)	0 (**100**)	0 (57.1)	0 (**100**)	0 (57.1)	0 (57.1)	0 (57.1)
SAGMCG-I	40	**95.0** (**95.0**)	**95.0** (**95.0**)	**97.5** (**97.5**)	**97.5** (**97.5**)	**97.5** (**97.5**)	7.5 (7.5)	**100** (**100**)			
SCG	355	**87.3** (**96.1**)	**88.2** (**96.9**)	**85.6** (**93.8**)	**87.6** (**96.3**)	**87.6** (**96.3**)	**83.4** (**91.5**)	**88.7** (**97.7**)			
UT-I	2	**100** (**100**)	**100** (**100**)	**100** (**100**)	50.0 (50.0)	50.0 (50.0)	**100** (**100**)	**100** (**100**)			
UT-II	1	**100** (**100**)	**100** (**100**)		**100** (**100**)	**100** (**100**)		**100** (**100**)			
UT-III	2	**100** (**100**)	**100** (**100**)	**100** (**100**)	**100** (**100**)	**100** (**100**)		**100** (**100**)			
DSAG	326	0 (**91.4**)	0 (**91.7**)	0 (12.3)	0 (**92.9**)	0 (**93.3**)	0 (0.9)	0 (**84.7**)	0 (3.1)	0 (3.1)	0 (3.1)
THSCG	50	0 (**100**)	0 (**98.0**)	0 (**92.0**)	0 (68.0)	0 (68.0)	0 (60.0)	0 (**94.0**)	0 (70.0)	0 (70.0)	0 (70.0)
MCG	483	0 (**88.0**)	0 (**88.2**)	0 (**93.8**)	0 (**92.3**)	0 (**93.2**)	0 (76.0)	0 (**88.6**)	0 (1.2)	0 (1.9)	0 (1.9)
UG	12	0 (58.3)	0 (58.3)	0 (**91.7**)	0 (**91.7**)	0 (**91.7**)	0 (**91.7**)	0 (**91.7**)			
Unclassified *Archaea*	272	0 (**88.6**)	0 (**89.3**)	0 (**84.6**)	0 (76.1)	0 (78.3)	0 (48.9)	0 (43.4)	0 (36.4)	0 (42.3)	0 (62.1)
Domain *Bacteria* c	667,899	(4.5 b)	(3.7)								

a Coverage values of universal primer.

b Coverage values of more than 80% for the target taxa are in bold, and tolerance values of more than 1% to the non-target taxa are under-lined.

c Estimation using RDP’s ProbeMatch.

Both primer pairs showed satisfactory specificity under experimental conditions, as predicted by our *in silico* analysis. Phylogenetic analyses of 436 and 423 clones from libraries constructed with THAUM-494-ARC917R and THAUM-494-1017FA showed that 95.5±3.7% and 96.9±6.4%, respectively, belonged to the phylum *Thaumarchaeota* (Table 6). The thaumarchaeotal subgroups most frequently sampled were SCG, MG-I, and SAGMCG-I. THAUM-494 showed very low non-target binding under the PCR conditions used in this study. The majority of non-target sequences amplified with both primer pairs belonged to the MCG subgroup. Interestingly, a considerable number of unclassified thaumarchaeotal sequences (UT sequences, 23–28%), whose phylogenetic positions within *Thaumarchaeota* were not precisely defined by our thaumarchaeotal subgrouping scheme, were observed in the Hillgate woodland soil sample and an Incheon paddy soil. These results suggest that *Thaumarchaeota* might contain additional as-yet-undiscovered diversity, which can be further explored with THAUM-494 in future work.

The proportions of thaumarchaeotal subgroups in each clone library varied slightly with the universal primer used. For example, MG-I sequences were not detected in the Kelleberrin sample when THAUM-494 was paired with 1017FAR; in contrast, MG-I sequences comprised 11% of the total amplified sequences when THAUM-494 was paired with ARC917R. We attributed such variations in subgroup detection to coverage differences between universal primers for each thaumarchaeotal subgroup. Our *in silico* analysis (Table 8) indicated that 1017FAR showed lower coverage (75.6%) for MG-I than ARC917R (96.0%). Hence, it is very important to pair THAUM-494 with the appropriate universal primer for unbiased sampling of *Thaumarchaeota* diversity. Although we experimentally tested only two universal primers to determine whether the choice of universal primer affects post-PCR sequence analysis, the coverages and tolerances of other well-known archaeal universal primers were estimated throughout the archaeal taxa by *in silico* analyses (Table 7 and Table 8; Table S5–S7). Thus, the results presented here will serve as a guideline for the selection of appropriate primer pairs for researchers to use in their particular applications.

### Concluding Remarks

Our knowledge of the phylum *Thaumarchaeota*, particularly regarding its ecological niche and diversity, is expanding rapidly, but is still limited. Since *Thaumarchaeota* are globally distributed and abundant [2], [62], these archaea likely play a crucial role in sustaining species diversity as well as maintaining geochemical cycles. Physiological, molecular, and ecological surveys have been undertaken to better understand this phylum. As a result of such efforts, a large number of thaumarchaeotal 16S rRNA gene sequences have been deposited in public databases. However, our rarefaction analyses indicate that the richness of thaumarchaeotal phylotypes has not yet reached its plateau, indicating that this phylum may have a much wider phylogenetic breadth than currently estimated. To facilitate comprehensive exploration of the diversity and ecological role of *Thaumarchaeota*, we developed THAUM-494, the first phylum-level primer for *Thaumarchaeota* to the best of our knowledge. The high coverage and low tolerance of THAUM-494 make it especially useful for estimating phylogenetic diversity and determining the distribution patterns of *Thaumarchaeota* (e.g., high-throughput metagenome sequencing and real-time PCR assays). Furthermore, this primer will be a valuable tool for understanding the ecological niche of *Thaumarchaeota*.

## Supporting Information

Figure S1 **Backbone phylogenetic tree.** The phylogenetic distances of each sequence were calculated using the Jukes-Cantor model, and the tree was constructed using the neighbor-joining algorithm. The numbers at the nodes indicates the bootstrap score (as a percentage) and are shown for the frequencies at or above the threshold of 50%. The scale bar represents the expected number of substitutions per nucleotide position.
(PDF)

Figure S2 **Phylogenetic positions of cloned sequences.** Cloned sequences recovered from Kellerberrin, Australia. A, primer pairs THAUM-494-ARC917R; B, primer pairs THAUM-494-1017R. The phylogenetic distances of each sequence were calculated using the Jukes-Cantor model, and the tree was constructed using the neighbor-joining algorithm. The numbers at the nodes indicates the bootstrap score (as a percentage) and are shown for the frequencies at or above the threshold of 50%. The scale bar represents the expected number of substitutions per nucleotide position.
(PDF)

Figure S3 **Phylogenetic positions of cloned sequences.** Cloned sequences recovered from Hillgate, California. A, primer pairs THAUM-494-ARC917R; B, primer pairs THAUM-494-1017R. The phylogenetic distances of each sequence were calculated using the Jukes-Cantor model, and the tree was constructed using the neighbor-joining algorithm. The numbers at the nodes indicates the bootstrap score (as a percentage) and are shown for the frequencies at or above the threshold of 50%. The scale bar represents the expected number of substitutions per nucleotide position.
(PDF)

Figure S4 **Phylogenetic positions of cloned sequences.** Cloned sequences recovered from La Campana, Chile. A, primer pairs THAUM-494-ARC917R; B, primer pairs THAUM-494-1017R. The phylogenetic distances of each sequence were calculated using the Jukes-Cantor model, and the tree was constructed using the neighbor-joining algorithm. The numbers at the nodes indicates the bootstrap score (as a percentage) and are shown for the frequencies at or above the threshold of 50%. The scale bar represents the expected number of substitutions per nucleotide position.
(PDF)

Figure S5 **Phylogenetic positions of cloned sequences.** Cloned sequences recovered from Incheon, Korea. A, primer pairs THAUM-494-ARC917R; B, primer pairs THAUM-494-1017R. The phylogenetic distances of each sequence were calculated using the Jukes-Cantor model, and the tree was constructed using the neighbor-joining algorithm. The numbers at the nodes indicates the bootstrap score (as a percentage) and are shown for the frequencies at or above the threshold of 50%. The scale bar represents the expected number of substitutions per nucleotide position.
(PDF)

Figure S6 **Phylogenetic positions of cloned sequences.** Cloned sequences recovered from Jeju, Korea. A, primer pairs THAUM-494-ARC917R; B, primer pairs THAUM-494-1017R. The phylogenetic distances of each sequence were calculated using the Jukes-Cantor model, and the tree was constructed using the neighbor-joining algorithm. The numbers at the nodes indicates the bootstrap score (as a percentage) and are shown for the frequencies at or above the threshold of 50%. The scale bar represents the expected number of substitutions per nucleotide position.
(PDF)

Table S1 **Sequences used for the backbone phylogenetic tree.**
(PDF)

Table S2 **Thaumarchaeotal sequences included in the local database.**
(PDF)

Table S3 **Primers designed in this study and their thermodynamic properties.**
(PDF)

Table S4 **Previously designed** ***Crenarchaeota*** **-directed primers and MG-I-directed primers not included in** **Table 5** **.**
(PDF)

Table S5 **Archaeal universal primers not included in** **Table 7** **.**
(PDF)

Table S6 ***In silico*** **evaluation of the specificity of the primers not included in** **Table 5** **and** **7** **(local database).**
(PDF)

Table S7 ***In silico*** **evaluation of the specificity of the primers not included in** **Table 5** **and** **7** **(RDP database).**
(PDF)
