# Correction: Novel PCR Primers for the Archaeal Phylum *Thaumarchaeota* Designed Based on the Comparative Analysis of 16S rRNA Gene Sequences

**DOI:** 10.1371/journal.pone.0175937

**Published:** 2017-04-11

**Authors:** Jin-Kyung Hong, Hye-Jin Kim, Jae-Chang Cho

Several of the primer sequences listed in Table 4 are incorrect. Please see the corrected Table 4 here.

**Table 4:** Primers used for PCR and *in silico* evaluation of the specificity.

Primer	Target taxon	Sequence (5'→3')	Sequence position		%GC	No. of degenerate sites	Thermodynamic properties^a^				Reference
			*E*. *coli*	*M*. *jannasch*			Rating	Tm	Hairpin ΔG	Dimer ΔG	
THAUM	Phylum *Thaumarchaeota*	GAATAAGGGGTGGGCAAGT	494–511	435–453	52.6	0	100	56.4	0.0	0.0	This study
CREN512	Phylum *Crenarchaeota*	CTGGTGTCAGCCGCCG	512–527	454–469	75.0	0	100	59.1	0.0	0.0	Jürgens et al., 2000
542F	Phylum *Crenarchaeota*	CGCGGTAATACCAGCYC	526–542	468–484	62.5	1	81	52.2	0.0	-10.4	Hershberger et al., 1996
Cren745a	Phylum *Crenarchaeota*	GGTGAGGGATGAAAGCTGGG	755–774	696–717	60.0	0	86	61.6	0.0	-7.3	Simon et al., 2000
Cren518R	Phylum *Crenarchaeota*	GCTGGTWTTACCGCGGCGGCTGA	518–541	460–482	68.2	1	68	74.6	-1.5	-16.5	Perevalova et al., 2003
GI-554	Group MG-I	AGGAKGATTATTGGGCCTAA	554–573	496–515	42.1	1	79	51.6	-1.2	-10.3	Massana et al., 1997
GI_751F	Group MG-I	GTCTACCAGAACAYGTTC	734–751	672–692	47.1	1	89	36.2	0.0	-5.9	Gubry-Rangin et al., 2010
771F	Group MG-I	ACGGTGAGGGATGAAAGCT	753–771	694–712	52.6	0	86	56.7	0.0	-7.3	Ochsenreiter et al., 2003
GI_956R	Group MG-I	HGGCGTTGACTCCAATTG	957–974	903–920	52.9	1	80	54.8	-0.6	-10.3	Beman et al., 2008
957R	Group MG-I	CGGCGTTGACTCCAATTG	957–974	903–920	55.6	0	83	57.7	-0.4	-10.3	Ochsenreiter et al., 2003
MCGI-554r^b^	Group MG-I	TGACCACTTGAGGTGCTG	537–554	479–496	55.6	0	92	51.8	-0.1	-4.3	Auguet et al., 2012
MCGI-391f	Group MG-I	AAGGTTARTCCGAGTGRTTTC	391–422	376–396	42.1	2	100	48.8	0.0	0.0	Auguet et al., 2012
333Fa^b^	Domain *Archaea*	TCCAGGCCCTACGGG	334–348	320–333	73.3	0	80	54.3	-1.9	-9.3	Baker et al., 2003
Arch338F	Domain *Archaea*	GGCCCTAYGGGGYGCASCAGGC	338–359	324–344	79.0	3	63	68.3	-4.8	-16.4	Kublanov et al., 2009
340RA	Domain *Archaea*	CCCCGTAGGGCCYGG	335–349	321–334	85.7	1	72	59.4	-2.6	-13.4	Barns et al., 1994
A340F	Domain *Archaea*	CCCTACGGGGYGCASCAG	340–357	326–342	75.0	2	97	56.9	-1.7	0.0	Vetriani et al., 1999
ARC349F	Domain *Archaea*	GYGCASCAGKCGMGAAW	349–365	334–350	66.7	5	100	39.4	0.0	0.0	Takai and Horikoshi, 2000
EK510R	Domain *Archaea*	CTTGCCCRGCCCTT	498–510	439–452	69.2	1	90.5	51.3	0.0	-5.1	Baker et al., 2003
ARC516	Domain *Archaea*	TGYCAGCCGCCGCGGTAAHACCVGC	516–541	458–482	72.7	3	55	78.9	-10.8	-16.5	Takai and Horikoshi, 2000
PARCH519F	Domain *Archaea*	CAGCMGCCGCGGTAA	519–533	461–475	71.4	1	68	55.2	0.0	-17.5	Øvreås et al., 1997
A751F	Domain *Archaea*	CCGACGGTGAGRGRYGAA	750–767	691–708	66.7	3	100	51.8	0.0	0.0	Baker et al., 2003
744RA	Domain *Archaea*	CCSGGGTATCTAATCC	785–800	726–741	53.3	1	76.5	46.8	0.0	-12.8	Barns et al., 1994
ARC806R	Domain *Archaea*	GGACTACVSGGGTATCTAAT	787–806	728–747	44.4	2	86.5	51.4	-0.8	-6.8	Takai and Horikoshi, 2000
765FA	Domain *Archaea*	TAGATACCCSSGTAGTCC	789–806	730–747	50.0	2	100	37.7	0.0	0.0	Barns et al., 1994
Ar9R	Domain *Archaea*	CCCGCCAATTCCTTTAAGTTTC	906–927	851–872	45.5	0	90	62.2	0.0	-5.4	Jürgens et al., 2000
ARC915R^b^	Domain *Archaea*	GTGCTCCCCCGCCAATTCCT	915–934	860–879	65.0	0	88	67.8	0.0	-6.4	Casamayor et al., 2000
ARC917R	Domain *Archaea*	GTGCTCCCCCGCCAATTC	915–934	860–879	66.7	0	88	63.2	0.0	-6.4	Loy et al., 2002
Arch958R^b^	Domain *Archaea*	YCCGGCGTTGAMTCCAATT	958–975	904–921	52.9	2	81	62.0	-0.4	-9.8	DeLong, 1992
1017FAR^b^	Domain *Archaea*	GGCCATGCACCWCCTCTC	1044–1060	982–999	70.6	1	83	58.3	0.0	-9.3	Barns et al., 1994
D34	Domain *Archaea*	GGTCTCGCTCGTTGCCTG	1096–1113	1035–1052	66.7	0	100	59.5	0.0	0.0	Arahal et al., 1996
A1098F	Domain *Archaea*	GGCAACGAGCGMGACCC	1098–1114	1037–1053	75.0	1	100	59.7	0.0	0.0	Baker et al., 2003
A1115R	Domain *Archaea*	GGGTCTCGCTCGTTG	1100–1114	1039–1053	66.7	0	100	48.5	0.0	0.0	Baker et al., 2003

^a^ Calculated using NetPrimer (http://www.premierbiosoft.com/netprimer). T_m_ was estimated using the Nearest neighbor method implemented in the NetPrimer.

^b^ Primers MCGI-554r, 333Fa, ARC915R, Arch958R, and 1017FAR are identical to CREN537, A333F, A934R, A976R, and A1040F, respectively.
