# Supplementary material for: Novel PCR Primers for the Archaeal Phylum Thaumarchaeota Designed Based on the Comparative Analysis of 16S rRNA Gene Sequences
Source: PLoS One. 2014 May 7;9(5):e96197. doi: 10.1371/journal.pone.0096197 (PMC4013054; doi:10.1371/journal.pone.0096197)
Supplement: Figure S1 — Backbone phylogenetic tree. The phylogenetic distances of each sequence were calculated using the Jukes-Cantor model, and the tree was constructed using the neighbor-joining algorithm. The numbers at the nodes indicates the bootstrap score (as a percentage) and are shown for the frequencies at or above the threshold of 50%. The scale bar represents the expected number of substitutions per nucleotide position. (PDF) [file pone.0096197.s001.pdf]

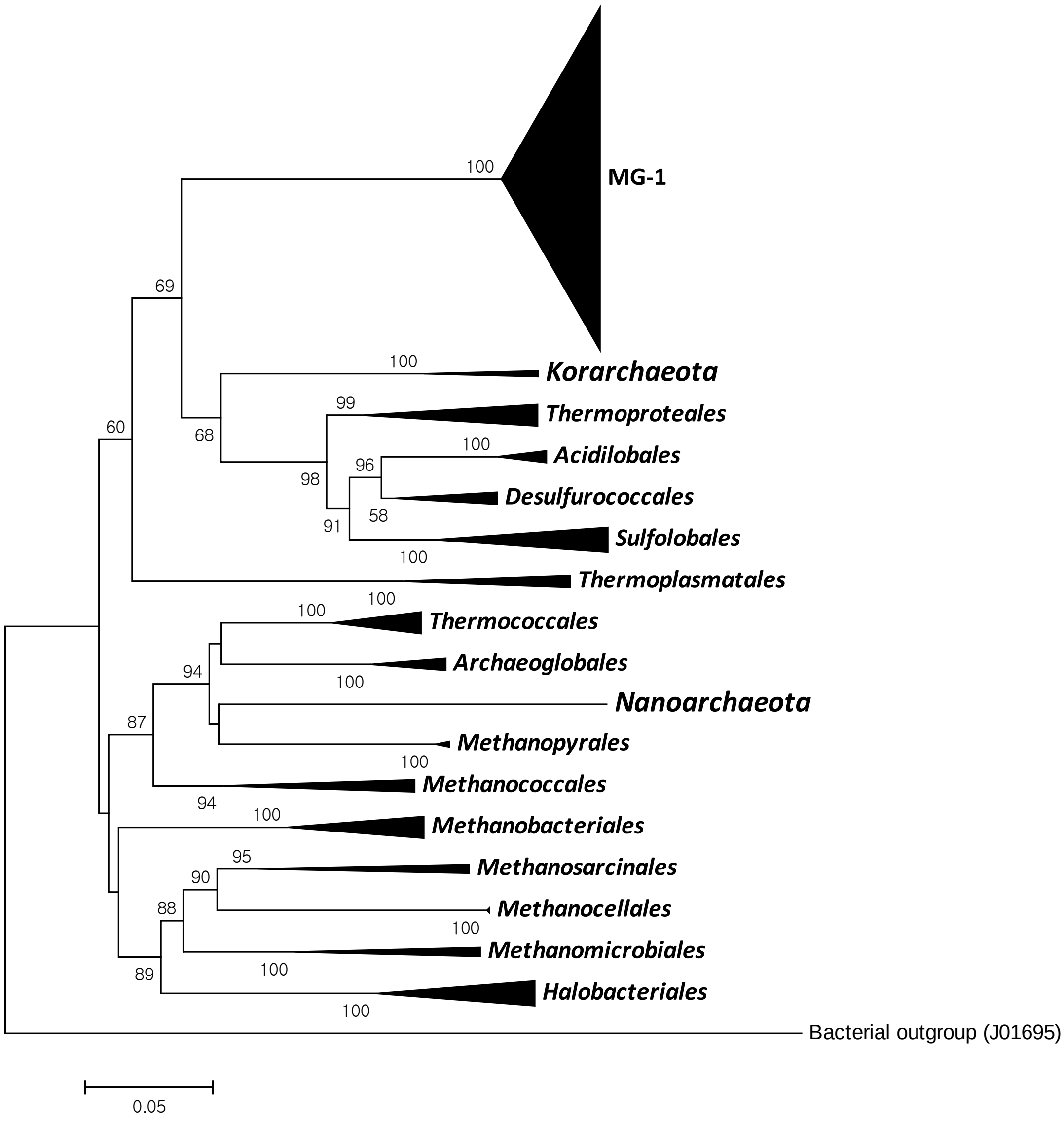

Supplementary Figure 1. Backbone phylogenetic tree showing the phylogenetic positions of the MG-I sequences and the representative sequences of archaeal taxa (Supplementary Table 1). The phylogenetic distances of each sequence were calculated using the Jukes-Cantor model, and the tree was constructed using the neighbor-joining algorithm. The numbers at the nodes indicates the bootstrap score (as a percentage) and are shown for the frequencies at or above the threshold of 50%. The scale bar represents the expected number of substitutions per nucleotide position.
