# Supplementary material for: Novel PCR Primers for the Archaeal Phylum Thaumarchaeota Designed Based on the Comparative Analysis of 16S rRNA Gene Sequences
Source: PLoS One. 2014 May 7;9(5):e96197. doi: 10.1371/journal.pone.0096197 (PMC4013054; doi:10.1371/journal.pone.0096197)
Supplement: Figure S4 — Phylogenetic positions of cloned sequences. Cloned sequences recovered from La Campana, Chile. A, primer pairs THAUM-494-ARC917R; B, primer pairs THAUM-494-1017R. The phylogenetic distances of each sequence were calculated using the Jukes-Cantor model, and the tree was constructed using the neighbor-joining algorithm. The numbers at the nodes indicates the bootstrap score (as a percentage) and are shown for the frequencies at or above the threshold of 50%. The scale bar represents the expected number of substitutions per nucleotide position. (PDF) [file pone.0096197.s004.pdf]

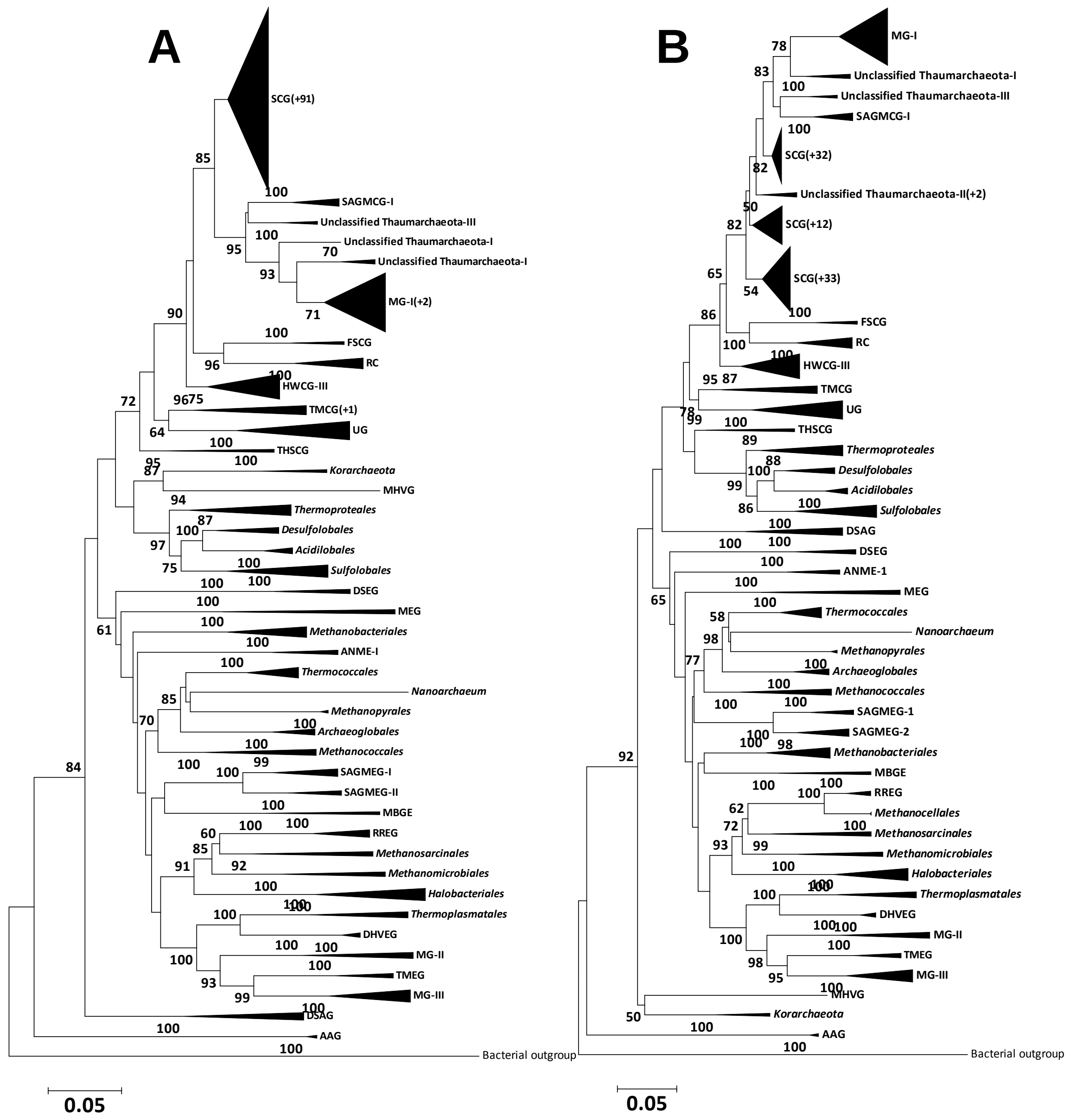

Supplementary Figure 4. Phylogenetic positions of cloned sequences recovered from La Campana (LC), Chile. A, primer pairs THAUM-494-ARC917R; B, primer pairs THAUM-494-1017R. The phylogenetic distances of each sequence were calculated using the Jukes-Cantor model, and the tree was constructed using the neighbor-joining algorithm. The numbers at the nodes indicates the bootstrap score (as a percentage) and are shown for the frequencies at or above the threshold of 50%. The scale bar represents the expected number of substitutions per nucleotide position.
