# Supplementary material for: Novel PCR Primers for the Archaeal Phylum Thaumarchaeota Designed Based on the Comparative Analysis of 16S rRNA Gene Sequences
Source: PLoS One. 2014 May 7;9(5):e96197. doi: 10.1371/journal.pone.0096197 (PMC4013054; doi:10.1371/journal.pone.0096197)
Supplement: Table S1 — Sequences used for the backbone phylogenetic tree. (PDF) [file pone.0096197.s007.pdf]

Table S1. Sequences used for the backbone phylogenetic tree.

Taxa			Sequences (GenBank accession numbers)									
Phylum	Class	Order										
Crenarchaeota	*Thermoprotei*	*Thermoproteales*	CP000505	CP000852	AE009441	CP000660	CP000561	CP000504	AB013926			
		Sulfolobales	CP000077	CP000682	D86414	D85506	D85520	AB010957	AY907891	X90482		
		Acidilobales	AF191225	D85038	EF057391	AY350586						
		Desulfolococcales	CP000575	X99562	AY264344	AJ012645						
Euryarchaeota	*Archaeoglobi*	*Archaeoglobales*	AE000782	CP001857	AF220166	AF220165						
	Halobacteria	*Halobacteriales*	CP001688	AM180088	CR936257	D14123	AB289741	EF645681	AB258305	D63572		
	Methanococci	*Methanococcales*	L77117	AF025822	AF056938	DQ195164						
	Methanobacteria	*Methanobacteriales*	CP001719	X99047	X15364	AF169245	X99044	DQ649335	U55240			
	Methanomicrobia	*Methanomicrobiales*	CP000559	CP000562	CP000254							
		Methanosarcinales	CP000477	CP000099	AE010299							
		Methanocellales	AP011532	AB196288								
	Methanopyri	*Methanopyrales*	AE009439	AB301476								
	Thermococci	*Thermococcales*	AE009950	BA000001	CP001398	AP006878	CP000855	AB019239	AY134472			
	Thermoplasmata	*Thermoplasmatales*	AE017261	AY907888	AJ224936	AB269873						
Korarchaeota			CP000968	AF255604								
Nanoarchaeota			AJ318041									
Thaumarchaeota			DQ085097	DQ085101	DQ085102	DQ085103	AF083072	AF083071	AF420236	AF420237	AF421159	AB050232
(MG-I)			AB050205	AF119126	AF119134	AF119138	AF119136	AF119127	AY627473	AY627472	AY627471	AY627467
			AY627466	AY627462	AY627461	AY627445	AY627444	AY627437	AY627433	AY627456	AY627469	EF106822
			EF106821	EF106817	EU309864	EU309861	EU309860	EU309865	FJ655771	FJ655769	FJ655767	FJ655763
			FJ655760	FJ655749	FJ655748	FJ655744	FJ655743	FJ655740	FJ655738	FJ655734	FJ655731	FJ655729
			FJ655726	FJ655723	FJ655697	FJ655695	FJ655691	FJ655690	FJ655688	FJ655682	FJ655679	FJ655666
			FJ655665	FJ655620	FJ655604	FJ655603	FJ655602	FJ655599	FJ655597	FJ655598	FJ655600	FJ655610
			FJ655616	FJ655618	FJ655619	FJ655651	FJ655663	FJ655664	FJ655669	FJ655677	FJ655684	FJ655685
			FJ655698	FJ655720	FJ655722	FJ655724	FJ655730	FJ655735	FJ655736	FJ655737	FJ655741	FJ655742
			FJ655747	FJ655751	FJ655754	FJ655756	FJ655757	FJ655758	FJ655761	FJ655762	FJ655768	FJ655770
			FJ810524	FJ810525	FJ810537	FJ810538	FJ971112	FJ971124				
