# Supplementary material for: Novel PCR Primers for the Archaeal Phylum Thaumarchaeota Designed Based on the Comparative Analysis of 16S rRNA Gene Sequences
Source: PLoS One. 2014 May 7;9(5):e96197. doi: 10.1371/journal.pone.0096197 (PMC4013054; doi:10.1371/journal.pone.0096197)
Supplement: Table S2 — Thaumarchaeotal sequences included in the local database. (PDF) [file pone.0096197.s008.pdf]

Table S2. Thaumarchaeotal sequences included in the local database.

Subgroups	Sequences (GenBank accession numbers)																			
FSCG	FJ468486	FJ468478	AB262709	AB243804	AB243803	AB243801	AB243794	AB243793	HM187532	HM187509	HM187479	HM187478	HM187470	HM187469	AB262710	AB262707	AB294269			
HWCG-III	GU137359	AB293212	GU137358	GU137363	GU137377	GU137378	GU137387	GU137390	GU137392	GU137393	AF361211	EU239960	EU635917	AB113623	AB201308	FJ821635	AB294267	AB213104	AB213105	EF032792
	GU137362	HM187468	HM187490	HM187515	HM187529															
MG-1	EU280202	AB019722	AB019723	AB019726	AB019727	AB019729	AB019730	AB050232	AB099992	AB193956	AB193958	AB193959	AB193960	AB193963	AB193964	AB193965	AB193967	AB193968	AB193971	AB193973
	AB193975	AB193982	AB193984	AB193986	AB193987	AB193988	AB193989	AB193993	AB193996	AB194003	AB194005	AB461944	AB461945	AB461949	AB461953	AB550810	AF119126	AF119134	AF119138	AF393304
	AF393305	AF393306	AF393307	AF419636	AF419646	AF420236	AF420237	AF421159	AJ347774	AY316120	AY505049	AY505050	AY505052	AY592231	AY592444	AY592448	AY592485	AY627456	AY627469	AY856355
	AY856356	AY856365	DQ299266	DQ299268	DQ299273	DQ299277	DQ299279	DQ299280	DQ300508	DQ300512	DQ300514	DQ300517	DQ300518	DQ300519	DQ300522	DQ300523	DQ300532	DQ300534	DQ300535	DQ300537
	DQ300539	DQ300540	DQ300542	DQ300543	DQ340995	DQ340996	DQ424907	DQ465920	DQ925860	EF069337	EF069338	EF069339	EF069342	EF069345	EF069349	EF069350	EF069354	EF069355	EF069356	EF069359
	EF069361	EF069362	EF069367	EF069369	EF069370	EF069371	EF069372	EF069378	EF069379	EF069380	EF069381	EF069382	EF076091	EF076092	EF076093	EF444625	EF597704	EF597717	EF645841	EF645842
	EF645843	EF645844	EF645845	EF645849	EF645850	EF645852	EF645853	EU280204	EU280216	EU280222	EU280224	EU280225	EU280226	EU280231	EU280232	EU283424	EU283425	EU284596	EU284597	EU284599
	EU284607	EU284613	EU284617	EU284620	EU284621	EU284624	EU284626	EU284629	EU284631	EU284634	EU284635	EU284639	EU284640	EU284641	EU284642	EU284644	EU284645	EU284646	EU284651	EU284655
	EU284656	EU284657	EU284666	EU309865	EU420675	EU420677	EU420687	EU420689	EU420704	EU420706	EU420709	EU686614	EU686615	EU686619	EU686623	EU686624	EU686633	EU686646	EU910614	FJ002863
	FJ002873	FJ002874	FJ150799	FJ150804	FJ150806	FJ150809	FJ150811	FJ150815	FJ150818	FJ150819	FJ150822	FJ150823	FJ150827	FJ150834	FJ487472	FJ487473	FJ487475	FJ487477	FJ487485	FJ487488
	FJ487490	FJ487491	FJ487494	FJ487495	FJ487498	FJ487499	FJ487500	FJ487501	FJ487504	FJ487505	FJ487506	FJ487509	FJ487516	FJ487521	FJ487523	FJ487524	FJ487525	FJ487527	FJ487529	FJ487532
	FJ487534	FJ487540	FJ487542	FJ487544	FJ487545	FJ487547	FJ487556	FJ487559	FJ487561	FJ571653	FJ571656	FJ571657	FJ571659	FJ571660	FJ571661	FJ571663	FJ571665	FJ571668	FJ571669	FJ571670
	FJ571673	FJ571674	FJ571677	FJ571688	FJ571690	FJ571693	FJ571694	FJ571700	FJ571702	FJ571705	FJ571706	FJ571709	FJ571710	FJ571715	FJ571717	FJ571718	FJ571719	FJ571720	FJ571722	FJ571724
	FJ571729	FJ571733	FJ571734	FJ571735	FJ571736	FJ571738	FJ571740	FJ571743	FJ571746	FJ571747	FJ571750	FJ571752	FJ571754	FJ571755	FJ571756	FJ571757	FJ571759	FJ571760	FJ571766	FJ571768
	FJ571770	FJ571773	FJ571774	FJ571775	FJ571778	FJ571779	FJ571780	FJ571782	FJ571783	FJ571784	FJ571785	FJ571786	FJ571788	FJ571789	FJ571791	FJ571792	FJ571793	FJ571795	FJ571796	FJ571797
	FJ571799	FJ571801	FJ571803	FJ571804	FJ571805	FJ571806	FJ571807	FJ571808	FJ571809	FJ571816	FJ571822	FJ571824	FJ571827	FJ571838	FJ571841	FJ571848	FJ571856	FJ571858	FJ571868	FJ571869
	FJ571870	FJ571872	FJ571878	FJ571882	FJ571883	FJ571884	FJ571885	FJ571888	FJ571891	FJ571895	FJ571896	FJ571897	FJ571898	FJ571899	FJ571901	FJ571902	FJ571903	FJ571904	FJ571906	FJ571911
	FJ571914	FJ571919	FJ571920	FJ571921	FJ571922	FJ571923	FJ571924	FJ571927	FJ571928	FJ571930	FJ571931	FJ571932	FJ571933	FJ571938	FJ571939	FJ571941	FJ571942	FJ571943	FJ571944	FJ571946
	FJ571947	FJ571948	FJ571949	FJ571951	FJ571952	FJ571954	FJ571958	FJ571961	FJ571963	FJ571966	FJ571967	FJ571968	FJ571969	FJ571970	FJ571971	FJ571972	FJ571974	FJ571975	FJ571979	FJ571981
	FJ571982	FJ571983	FJ571984	FJ571985	FJ655598	FJ655600	FJ655610	FJ655616	FJ655618	FJ655619	FJ655651	FJ655663	FJ655664	FJ655669	FJ655677	FJ655684	FJ655685	FJ655698	FJ655720	FJ655722
	FJ655724	FJ655730	FJ655735	FJ655736	FJ655737	FJ655741	FJ655742	FJ655747	FJ655751	FJ655754	FJ655756	FJ655757	FJ655758	FJ655761	FJ655762	FJ655768	FJ655770	FJ718906	FJ718907	FJ718909
	FJ718910	FJ718916	FJ718917	FJ718918	FJ718920	FJ718922	FJ718925	FJ718926	FJ718927	FJ718930	FJ718931	FJ718932	FJ718933	FJ718934	FJ718935	FJ718937	FJ718938	FJ718939	FJ718940	FJ718941
	FJ718942	FJ718943	FJ718945	FJ718946	FJ718947	FJ718949	FJ718950	FJ718952	FJ718953	FJ718955	FJ718961	FJ718962	FJ718967	FJ718970	FJ718971	FJ718973	FJ718977	FJ718978	FJ718982	FJ718985
	FJ752631	FJ752632	FJ810524	FJ971109	FJ971110	FJ971111	FJ971112	FJ971113	FJ971114	FJ971116	FJ971118	FJ971120	FJ971124	FJ971125	FJ981534	FN421233	FN820356	GU137354	GU137367	GU137368
	GU137380	GU137381	GU137382	GU137391	GU363064	GU386315	GU553442	GU553802	GU553809	GU553811	U51469	U71110	U71112	HM187506	GU553818	GU553817	GU553815	GU553812	GU553810	GU553808
	GU553807	GU553806	GU553803	GU553800	GU553440	GU553435	FN820422	FN820415	FN820358	AB546961	AB453932	EU420707	EU420691	EU420688	EU420686	EU420685	EU420679	EU420678	EU420674	GU363083
	GU363068	GU363067	GU363065	AB550808	AB550807	GU137385	GU137384	GU137383	GU137376	GU137375	GU137373	GU137372	GU137371	GU137370	GU137369	GU137360	GU137357	GU137356	GU137355	GU137353
	FJ718987	FJ718984	FJ718983	FJ718981	FJ718980	FJ718979	FJ718976	FJ718975	FJ718974	FJ718972	FJ718969	FJ718968	FJ718966	FJ718965	FJ718964	FJ718963	FJ718960	FJ718959	FJ718958	FJ718957
	FJ718956	FJ718954	FJ718951	FJ718948	FJ718944	FJ718936	FJ718929	FJ718928	FJ718924	FJ718923	FJ718921	FJ718919	FJ718915	FJ718914	FJ718913	FJ718912	FJ718911	FJ718908	FJ971126	FJ971123
	FJ971122	FJ971121	FJ971119	FJ971117	FJ971115	FJ971108	FJ810525	FJ752633	FJ655771	FJ655769	FJ655767	FJ655763	FJ655760	FJ655749	FJ655748	FJ655744	FJ655743	FJ655740	FJ655738	FJ655734
	FJ655731	FJ655729	FJ655726	FJ655723	FJ655697	FJ655695	FJ655691	FJ655690	FJ655688	FJ655682	FJ655679	FJ655666	FJ655665	FJ655620	FJ655604	FJ655603	FJ655602	FJ655599	FJ655597	U78199
	AY592543	AY592516	AY592439	AY592437	FJ571980	FJ571978	FJ571977	FJ571976	FJ571973	FJ571965	FJ571964	FJ571962	FJ571960	FJ571959	FJ571957	FJ571956	FJ571955	FJ571953	FJ571950	FJ571945
	FJ571940	FJ571937	FJ571936	FJ571935	FJ571934	FJ571929	FJ571926	FJ571925	FJ571918	FJ571917	FJ571916	FJ571915	FJ571913	FJ571910	FJ571909	FJ571908	FJ571907	FJ571905	FJ571900	FJ571894
	FJ571893	FJ571892	FJ571890	FJ571889	FJ571887	FJ571886	FJ571881	FJ571880	FJ571879	FJ571877	FJ571876	FJ571875	FJ571874	FJ571873	FJ571871	FJ571867	FJ571866	FJ571865	FJ571864	FJ571863
	FJ571862	FJ571861	FJ571860	FJ571859	FJ571857	FJ571855	FJ571854	FJ571853	FJ571852	FJ571851	FJ571850	FJ571849	FJ571847	FJ571846	FJ571845	FJ571844	FJ571843	FJ571842	FJ571840	FJ571839
	FJ571837	FJ571836	FJ571835	FJ571834	FJ571833	FJ571832	FJ571831	FJ571830	FJ571829	FJ571828	FJ571826	FJ571825	FJ571823	FJ571821	FJ571820	FJ571819	FJ571818	FJ571817	FJ571815	FJ571814
	FJ571813	FJ571812	FJ571811	FJ571810	FJ571802	FJ571800	FJ571798	FJ571794	FJ571790	FJ571787	FJ571781	FJ571777	FJ571776	FJ571772	FJ571771	FJ571769	FJ571767	FJ571765	FJ571764	FJ571763
	FJ571762	FJ571761	FJ571758	FJ571753	FJ571751	FJ571749	FJ571748	FJ571745	FJ571744	FJ571742	FJ571741	FJ571739	FJ571737	FJ571732	FJ571731	FJ571730	FJ571728	FJ571727	FJ571726	FJ571725
	FJ571723	FJ571721	FJ571716	FJ571714	FJ571713	FJ571712	FJ571711	FJ571708	FJ571707	FJ571704	FJ571703	FJ571701	FJ571699	FJ571698	FJ571697	FJ571696	FJ571695	FJ571692	FJ571691	FJ571689
	FJ571687	FJ571686	FJ571685	FJ571684	FJ571683	FJ571682	FJ571681	FJ571680	FJ571679	FJ571678	FJ571676	FJ571675	FJ571672	FJ571671	FJ571667	FJ571666	FJ571664	FJ571662	FJ571658	FJ571652
	FJ526997	FJ487563	FJ487562	FJ487560	FJ487558	FJ487557	FJ487555	FJ487554	FJ487553	FJ487552	FJ487551	FJ487550	FJ487549	FJ487548	FJ487546	FJ487543	FJ487541	FJ487539	FJ487538	FJ487537
	FJ487536	FJ487535	FJ487533	FJ487531	FJ487530	FJ487528	FJ487526	FJ487522	FJ487520	FJ487519	FJ487518	FJ487517	FJ487515	FJ487514	FJ487513	FJ487512	FJ487511	FJ487510	FJ487508	FJ487507
	FJ487503	FJ487502	FJ487497	FJ487493	FJ487492	FJ487489	FJ487486	FJ487484	FJ487483	FJ487482	FJ487481	FJ487480	FJ487479	FJ487478	FJ487476	FJ487474	AB461952	AB461950	AB461948	AB461947
	AB461946	AB461943	AB461942	FM179919	FJ150833	FJ150832	FJ150831	FJ150830	FJ150829	FJ150828	FJ150826	FJ150825	FJ150824	FJ150821	FJ150820	FJ150817	FJ150813	FJ150812	FJ150810	FJ150807
	FJ150801	FJ150800	FJ150797	FJ150796	FJ150794	FJ002876	FJ002868	FJ002866	FJ002862	EU910613	EU686642	EU283427	EU283426	EU283423	EU686625	U71114	EU650267	EU650253	EU650252	EU650244
	EU650239	EU650237	AY627473	AY627472	AY627471	AY627467	AY627466	AY627462	AY627461	AY627445	AY627444	AY627437	AY627433	CP000866	EU309864	EU309861	EU309860	EU284662	EU284661	EU284660
	EU284659	EU284654	EU284652	EU284647	EU284643	EU284638	EU284636	EU284633	EU284632	EU284630	EU284628	EU284627	EU284625	EU284623	EU284622	EU284618	EU284616	EU284603	EU284602	EU284601
	EU280234	EU280229	EU280228	EU280227	EU280208	EU280207	EU280206	EU280203	EU280201	EF597707	AB329817	AB329802	AB329773	EF645854	EF645851	EF645848	EF645847	EF645846	EF645840	EF645839
	EF645838	EF076167	EF076166	EF076165	AY591971	AY591948	AY591939	AY591933	EF444675	EF444653	EF106822	EF106821	EF106817	AB294266	AB294264	AB293246	EF069388	EF069386	EF069385	EF069384
	EF069383	EF069377	EF069376	EF069375	EF069374	EF069373	EF069368	EF069366	EF069365	EF069364	EF069363	EF069360	EF069358	EF069357	EF069353	EF069352	EF069351	EF069348	EF069347	EF069346
	EF069344	EF069343	EF069341	EF069340	DQ659409	DQ536433	U71118	U71117	U71116	U71115	U71113	U71111	U71109	DQ299278	DQ299272	DQ300541	DQ300538	DQ300536	DQ300533	DQ300531
	DQ300530	DQ300529	DQ300528	DQ300526	DQ300525	DQ300524	DQ300521	DQ300520	DQ300516	DQ300515	DQ300513	DQ300511	DQ300510	DQ300509	DQ085103	DQ085102	DQ085101	DQ085097	AB194004	AB194002
	AB194001	AB194000	AB193999	AB193992	AB193983	AB193981	AB193980	AB193979	AB193974	AB193972	AB193970	AB193969	AB193957	AB019731	AB019728	AB019725	AB019724	U40238	AF393466	AF083072
	AF083071	AJ535118	AJ347777	AJ347776	AJ347775	AY505054	AY505053	AY505051	AY505048	AY505047	AY505046	AY082453	AY082452	AF119136	AF119127	FJ571912	FJ487496	EF069387	AB050205	AB294265
	DQ133423	FJ810537	FJ810538	HM187535	AB550817	AB550816	AB550815	AB550814	AB550813	AB550809	FJ971897	FJ971896	FJ971895	AB050238	AB113625	AB050207	AB050234	AB193991	DQ133425	FJ487487
RC	AB262708	AB243795	DQ223194	DQ223193	DQ223192	DQ223190	DQ223188													
SAGMCG-1	AB550812	FJ810535	EU309869	EU309868	EU309859	DQ837284	DQ837281	AB294263	AB294261	EF032791	AB243811	AB243809	AB243807	AB243798	AB243797	DQ223191	DQ223189	AB050241	AB050240	AB050229
	AB050227	AB050208	AF389433	EF032790	HM187516	HM187555	HM187557	HM187570	AB243800	AB294259	AB294262	AB550811	EU309863	EU309867	GU135485	HM187548	HM187547	HM187544	HM187518	AB294260
SCG	HM187572	HM187567	HM187566	HM187565	HM187564	HM187561	HM187559	HM187558	HM187539	HM187536	HM187534	HM187533	HM187530	HM187526	HM187525	HM187522	HM187520	HM187517	HM187514	HM187463
	HM187461	HM187459	HM187457	AB541973	AB541972	AB541971	AB541970	AB541969	AB541968	AB541967	AB541966	AB541965	AB541964	AB541963	AB541962	AB541961	AB541960	AB541959	AB541958	AB541957
	AB541956	AB541955	AB541954	AB541923	AB541922	AB541921	AB541920	AB541919	AB541918	AB541917	AB541916	AB541915	AB541914	AB541913	AB541912	AB541911	AB541910	AB541909	AB541908	AB541907
	AB541906	AB541905	AB541904	AB541903	AB541902	AB541901	AB541900	AB541899	AB541898	AB541897	AB541870	AB541856	AB541855	AB541854	AB541853	AB541852	AB541851	AB541850	AB541849	AB541848
	AB541847	AB541846	AB541845	AB541844	AB541843	AB541842	AB541841	AB541840	AB541839	AB541838	AB541820	AB541819	AB541818	AB541817	AB541815	AB541814	AB541762	AB541761	AB541760	AB541759
	AB541758	AB541757	AB541756	AB541755	AB541754	AB541753	AB541752	AB541751	AB541749	AB541733	AB541732	AB541694	AB541693	AB541692	AB541691	AB541690	AB541689	AB541688	AB541687	AB541686
	AB541685	AB541684	AB541683	AB541682	AB541638	AB541619	AB541617	AB541591	AB541589	FJ982761	FJ982759	FJ982757	FJ982756	FJ790598	FJ790597	FJ790595	FJ790594	FJ790593	FJ790592	FJ790591
	FJ790590	FJ790589	FJ790588	FJ790536	FJ790535	FJ790534	FJ790533	FJ790532	FJ790531	FJ790530	FJ790529	FJ790528	FJ790527	FJ790526	FJ790525	FJ790524	FJ790523	FJ790522	FJ790521	FJ790520
	FJ790519	FJ790518	FJ790517	FJ784314	FJ784313	FJ784312	FJ784311	FJ784310	FJ784309	FJ784308	FJ784307	FJ784305	FJ784304	FJ784303	FJ784302	FJ784301	FJ784300	FJ784299	FJ784298	FJ784297
	FJ784296	FJ784295	FJ784294	FJ784293	FJ784292	FJ784291	FJ784290	FJ784289	FJ784288	FJ784287	FJ784286	FJ784285	FJ784284	FJ784283	FJ784282	FJ784281	FJ784280	FJ784279	FJ784278	FJ784277
	FJ784276	FJ784275	FJ784274	FJ784273	FJ784272	FJ784271	FJ784270	FJ784269	FJ784268	FJ784267	FJ784266	FJ784265	FJ784264	FJ784263	FJ784262	FJ784261	FJ784260	FJ784259	FJ784258	FJ784257
	FJ784256	FJ784255	FJ784254	FJ784253	FJ784252	FJ784251	FJ784250	FJ784249	FJ784248	FJ784247	FJ784246	FJ784244	FJ784243	FJ784242	FJ705104	FJ655668	U62820	U62819	U62818	U62817
	U62816	U62815	U62814	U62813	U62812	U62811	FJ468484	FJ468483	FJ468482	FJ468481	FJ468480	FJ468479	FJ468477	EU281336	EU281335	EU281334	EU281333	EU281332	EU309866	EU309862
	EU284637	EU284619	EU284615	EU284612	EU284611	EU284610	EU284609	EU284606	EU284600	EU280230	EU155998	EU155997	EF562195	EF562193	EF562191	EF562189	EF690623	EF690622	EF032794	EF032793
	AY487104	AY487103	AY487102	AY487101	AY487100	AB050233	AB050230	AB050228	AB050221	AJ627422	AJ535123	AJ535122	AJ535121	AJ535120	AJ535119	AJ535117	AY522902	AY522901	AY522900	AY522899
	AY522898	AY522897	AY522896	AY522895	AY522894	AY522893	AY522892	AY522891	AY522890	AY522889	AY522888	AY522887	AY522886	AY522885	AY522884	AY522883	AY522882	AY522881	AY522880	AY522879
	AY522878	AY522877	AY522876	AY522875	AY522874	AY522873	AY522872	AY522871	AY522870	AY522869	AY522868	AY522867	AY522866	AY522865	AY522864	AY522863	AY522862	AY522861	AY278106	U59968
	AF227644	AF227643	AF227642	AF227641	AF227640	AF227639	AF227638	AF227637	AF227636	AF227635	AB113633	AB113630	AB113626	AB294268	HM187524					
UT-I	AB050231	AB113628																		
UT-II	EF444677																			
UT-III	HM187528	HM187541																		
