# Supplementary material for: Novel PCR Primers for the Archaeal Phylum Thaumarchaeota Designed Based on the Comparative Analysis of 16S rRNA Gene Sequences
Source: PLoS One. 2014 May 7;9(5):e96197. doi: 10.1371/journal.pone.0096197 (PMC4013054; doi:10.1371/journal.pone.0096197)
Supplement: Table S3 — Primers designed in this study and their thermodynamic properties. (PDF) [file pone.0096197.s009.pdf]

Table S3. Primers designed in this study and their thermodynamic properties. Primer N-10 corresponds to the primer THAUM-494 described in the text.

Primer	Sequence (5'→3')	Sequence position		%GC	No. of degenerate sites	Thermodynamic properties[a]			
		E. coli	*M. jannasch*			Rating	Tm	Hairpin ΔG	Dimer ΔG
N-08	CCTATGGGGCGCAGCAGGC	340-359	326-344	73.7	0	81	68.2	-0.8	-9.9
N-10	GAATAAGGGGTGGGCAAGT	494-511	435-453	52.6	0	100	56.4	0.0	0.0
S-04	TGGTGAGGTAATGGCC	253-268	239-254	56.3	0	81	48.3	0.0	-10.3
S-05	GGTGAGGTAATGGCCC	255-270	241-256	62.5	0	81	49.9	0.0	-10.3
S-06	GTGAGGTAATGGCCCA	254-269	240-255	56.3	0	81	48.3	0.0	-10.3
S-07	TGAGGTAATGGCCCAC	257-272	243-258	56.3	0	81	48.3	0.0	-10.3
S-08	GAGGTAATGGCCCACC	256-271	242-257	62.5	0	81	49.9	-1.3	-9.3
S-09	AGGTAATGGCCCACCA	258-273	244-259	56.3	0	81	51.0	-1.5	-9.3
S-11	CCTATGGGGCGCAGCA	340-356	326-341	68.8	0	82	58.9	0.0	-9.9
S-12	CTATGGGGCGCAGCAG	341-357	327-342	68.8	0	82	55.8	0.0	-9.9
S-13	TATGGGGCGCAGCAGG	343-358	328-343	68.8	0	82	58.9	0.0	-9.9
S-14	ATGGGGCGCAGCAGGC	344-359	329-344	75.0	0	82	63.1	0.0	-9.9
S-19	TAAGGGGTGGGCAAGT	497-511	438-453	56.3	0	100	50.5	0.0	0.0

[a] Calculated using NetPrimer (http://www.premierbiosoft.com/netprimer). T_m was estimated using the Nearest neighbor method implemented in the NetPrimer.
