# Supplementary material for: Novel PCR Primers for the Archaeal Phylum Thaumarchaeota Designed Based on the Comparative Analysis of 16S rRNA Gene Sequences
Source: PLoS One. 2014 May 7;9(5):e96197. doi: 10.1371/journal.pone.0096197 (PMC4013054; doi:10.1371/journal.pone.0096197)
Supplement: Table S4 — Previously designed Crenarchaeota -directed primers and MG-I-directed primers not included in Table 5 . (PDF) [file pone.0096197.s010.pdf]

Table S4. Previously designed *Crenarchaeota*-directed primers and MG-I-directed primers not included in Table 5 due to the low coverage for the phylum *Thaumarchaeota* or high tolerance to the non-thaumarchaeotal taxa. Target taxa are shown as the name at the time of the primer design.

Primer	Target taxon	Sequence (5'→3')	Sequence position		%GC	No. of degenerate sites	Thermodynamic properties[a]				Reference
			E. coli	*M. jannasch*			Rating	Tm	Hairpin ΔG	Dimer ΔG	
89F	Group MG-I	GGCTCAGTAACGCGTAGTC	111-129	91-109	57.9	0	76	53.1	0.0	-13.1	Hershberger et al., 1996
G-IV-1F	Group MG-I	TAATAACAGATAGTACTCCT	115-133	95-114	30.0	0	85	38.0	0.0	-7.8	López-García et al., 2001
Cren28F	Phylum *Crenarchaeota*	AATCCGGTTGATCCTGCCGGACC	6-28	1-23	60.9	0	66	71.9	-8.1	-45.6	Schleper et al., 1997
Cren7F	Phylum *Crenarchaeota*	TTCCGGTTGATCCYGCCGGACC	7-28	2-23	66.7	1	66	72.6	-8.2	-12.3	Perevalova et al., 2003
89Fb	Phylum *Crenarchaeota*	ACGGCTCAGTAACRC	109-123	89-103	57.1	1	100	37.2	0.0	0.0	Buckley et al., 1998
Cren113a	Phylum *Crenarchaeota*	CTCAGTAACACGTAGTCAACAT	113-133	93-114	40.9	0	88	49.6	0.0	-6.3	Simon et al., 2000
Cren113	Phylum *Crenarchaeota*	TCAGTAACACGTAGTCAACAT	114-133	94-114	38.1	0	88	47.6	0.0	-6.3	Simon et al., 2000
Cren457R	Phylum *Crenarchaeota*	CGGGSGAATAAGCGGGGGGCAA	489-509	432-453	66.7	1	100	73.4	0.0	0.0	Schleper et al., 1997
Cren499	Phylum *Crenarchaeota*	GGTCTGAACGGGGGGCGA	493-509	434-451	72.2	0	100	65.9	0.0	0.0	Burggraf et al., 1994
Cren518R	Phylum *Crenarchaeota*	TCAGCCGCCGCGGTAAWACCAGC	518-540,541	460-482	68.2	1	68	74.7	-1.5	-16.5	Perevalova et al., 2003
CREN569	Phylum *Crenarchaeota*	CCTAAAGCATCCGTAGC	569-585	511-527	52.9	0	93	48.3	0.0	-3.6	Jurgens et al., 2000
Cren1209	Phylum *Crenarchaeota*	CCGAAACCCTCGGGCCAC	1209-1226	1155-1172	72.2	0	76	65.5	-3.9	-10.3	Simon et al., 2000

[a] Calculated using NetPrimer (http://www.premierbiosoft.com/netprimer). T_m was estimated using the Nearest neighbor method implemented in the NetPrimer.

References

1. Hershberger KL, Barns SM, Reysenbach AL, Dawson SC, Pace NR (1996) Wide diversity of Crenarchaeota. Nature 384: 420.
2. López-García P, Moreira D, Lopez-Lopez A, Rodriguez-Valera F (2001) A novel haloarchaeal-related lineage is widely distributed in deep oceanic regions. Environ Microbiol 3: 72-78.
3. Schleper C, Holben W, Klenk HP (1997) Recovery of crenarchaeotal ribosomal DNA sequences from freshwater-lake sediments. Appl Environ Microbiol 63: 321-323.
4. Perevalova AA, Kolganova TV, Birkeland NK, Schleper C, Bonch-Osmolovskaya EA, et al. (2008) Distribution of Crenarchaeota representatives in terrestrial hot springs of Russia and Iceland. Appl Environ Microbiol 74: 7620-7628.
5. Buckley DH, Graber JR, Schmidt TM (1998) Phylogenetic analysis of nonthermophilic members of the kingdom crenarchaeota and their diversity and abundance in soils. Appl Environ Microbiol 64: 4333-4339.
6. Simon HM, Dodsworth JA, Goodman RM (2000) Crenarchaeota colonize terrestrial plant roots. Environ Microbiol 2: 495-505.
7. Burggraf S, Mayer T, Amann R, Schadhauser S, Woese CR, et al. (1994) Identifying members of the

domain Archaea with rRNA-targeted oligonucleotide probes. Appl Environ Microbiol 60: 3112-3119.

8. Jurgens G, Glöckner F, Amann R, Saano A, Montonen L, et al. (2000) Identification of novel Archaea in bacterioplankton of a boreal forest lake by phylogenetic analysis and fluorescent in situ hybridization. FEMS Microbiol Ecol 34: 45-56.
