# Supplementary material for: Novel PCR Primers for the Archaeal Phylum Thaumarchaeota Designed Based on the Comparative Analysis of 16S rRNA Gene Sequences
Source: PLoS One. 2014 May 7;9(5):e96197. doi: 10.1371/journal.pone.0096197 (PMC4013054; doi:10.1371/journal.pone.0096197)
Supplement: Table S5 — Archaeal universal primers not included in Table 7 . (PDF) [file pone.0096197.s011.pdf]

Table S5. Archaeal universal primers not included in Table 7.

Primer	Sequence (5'→3')	Sequence position		%GC	No. of degenerate sites	Thermodynamic properties[a]				Reference
		E. coli	*M. jannasch*			Rating	Tm	Hairpin ΔG	Dimer ΔG	
D30[b]	ATTCCGGTTGATCCTGC	6-22	1-17	52.9	0	82	52.9	0.0	-9.8	Arahal et al., 1996
21F[b]	TTCCGGTTGATCCYGCCGGA	7-26	2-21	63.2	1	66	68.7	-8.2	-12.3	DeLong, 1992
A3Fa	TCCGGTTGATCCYGCCGG	8-25	3-20	70.6	1	71	65.4	-6.7	-10.8	McInerney et al., 1995
A3Fb	TCYGKTTGATCCYGSCRGAG	8-27	3-22	60.0	5	76	50.3	-2.1	-11.4	López-García et al., 2001
23F	TCYGGTTGATCCTGCC	8-23	3-18	60.0	1	91	48.6	0.0	-4.6	Burggraf et al., 1994
A25F	CYGGTTGATCCTGCCRG	9-25	4-20	66.7	2	81	52.8	-3.5	-7.7	Dojka et al., 1998
Arch69F	TAAGCCATGCAAGTCGACG	49-67	45-63	52.6	0	81	57.8	0.0	-10.5	Tokura et al., 2000
A109F	ACKGCTCAGTAACACGT	109-125	89-105	50.0	1	84	41.7	-1.7	-7.3	Whitehead and Cotta, 1999
D33	ACGGGGCGCAGGCGCGA	344-360	329-345	82.4	0	68	73.2	-7.8	-11.4	Arahal et al., 1996
A344F[b]	ACGGGGTGCAGCAGGCGCGA	344-363	329-348	75.0	0	79	75.4	0.0	-11.4	Casamayor et al., 2000
Kb366F	CTCCGCAATRCGCGMAAG	367-384	352-369	62.5	2	69	56.0	-3.3	-14.5	Brunk and Eis, 1998
W034	GTGCCAGCAGCCGCGGTAA	515-533	457-475	68.4	0	68	67.5	0.0	-17.5	Leclerc et al., 2001
UA1204R	AGGTMNGYATGCCCCKAA	1191-1208	1137-1154	57.1	4	100	45.0	0.0	0.0	Baker et al., 2003
EKb1242R	CACGCGSGCTACAATGG	1221-1237	1167-1183	62.5	1	81	53.7	0.0	-10.4	Baker et al., 2003
Arch1381R	CCCTGCYCCTTGCACACACCGC	1381-1402	1327-1348	71.4	1	86	72.2	-0.6	-7.1	Kublanov et al., 2009
arc1492r	AAGTCGTAACAAGGTAGCC	1492-1510	1429-1447	47.4	0	100	49.3	0.0	0.0	Teske et al., 2002

[a] Calculated using NetPrimer (http://www.premierbiosoft.com/netprimer). T_m was estimated using the Nearest neighbor method implemented in the NetPrimer.

[b] Primers D30 and A344F are identical to A1F and Arc344F, respectively, and 21F is identical to A2Fa and A2Fb.

References

1. Arahal DR, Dewhirst FE, Paster BJ, Volcani BE, Ventosa A (1996) Phylogenetic analyses of some extremely halophilic archaea isolated from Dead Sea water, determined on the basis of their 16S rRNA sequences. Appl Environ Microbiol 62: 3779-3786.
2. DeLong EF (1992) Archaea in coastal marine environments. Proc Natl Acad Sci U S A 89: 5685-5689.
3. McInerney JO, Wilkinson M, Patching JW, Embley TM, Powell R (1995) Recovery and phylogenetic analysis of novel archaeal rRNA sequences from a deep-sea deposit feeder. Appl Environ Microbiol 61: 1646-1648.
4. López-García P, Moreira D, Lopez-Lopez A, Rodriguez-Valera F (2001) A novel haloarchaeal-related lineage is widely distributed in deep oceanic regions. Environ Microbiol 3: 72-78.
5. Burggraf S, Mayer T, Amann R, Schadhauser S, Woese CR, et al. (1994) Identifying members of the domain Archaea with rRNA-targeted oligonucleotide probes. Appl Environ Microbiol 60: 3112-3119.

6. Dojka MA, Hugenholtz P, Haack SK, Pace NR (1998) Microbial diversity in a hydrocarbon- and chlorinated-solvent-contaminated aquifer undergoing intrinsic bioremediation. Appl Environ Microbiol 64: 3869-3877.

7. Tokura M, Ohkuma M, Kudo T (2000) Molecular phylogeny of methanogens associated with flagellated protists in the gut and with the gut epithelium of termites. FEMS Microbiol Ecol 33: 233-240.

8. Whitehead TR, Cotta MA (1999) Phylogenetic diversity of methanogenic archaea in swine waste storage pits. FEMS Microbiol Lett 179: 223-226.

9. Casamayor EO, Massana R, Benlloch S, Ovreas L, Diez B, et al. (2002) Changes in archaeal, bacterial and eukaryal assemblages along a salinity gradient by comparison of genetic fingerprinting methods in a multipond solar saltern. Environ Microbiol 4: 338-348.

10. Brunk CF, Eis N (1998) Quantitative measure of small-subunit rRNA gene sequences of the kingdom korarchaeota. Appl Environ Microbiol 64: 5064-5066.

11. Leclerc M, Delbes C, Moletta R, Godon J (2001) Single strand conformation polymorphism monitoring of 16S rDNA Archaea during start-up of an anaerobic digester. FEMS Microbiol Ecol 34: 213-220.

12. Baker GC, Smith JJ, Cowan DA (2003) Review and re-analysis of domain-specific 16S primers. J Microbiol Methods 55: 541-555.

13. Kublanov IV, Perevalova AA, Slobodkina GB, Lebedinsky AV, Bidzhieva SK, et al. (2009) Biodiversity of thermophilic prokaryotes with hydrolytic activities in hot springs of Uzon Caldera, Kamchatka (Russia). Appl Environ Microbiol 75: 286-291.

14. Teske A, Hinrichs KU, Edgcomb V, de Vera Gomez A, Kysela D, et al. (2002) Microbial diversity of hydrothermal sediments in the Guaymas Basin: evidence for anaerobic methanotrophic communities. Appl Environ Microbiol 68: 1994-2007.
