# Supplementary material for: Novel PCR Primers for the Archaeal Phylum Thaumarchaeota Designed Based on the Comparative Analysis of 16S rRNA Gene Sequences
Source: PLoS One. 2014 May 7;9(5):e96197. doi: 10.1371/journal.pone.0096197 (PMC4013054; doi:10.1371/journal.pone.0096197)
Supplement: Table S6 — In silico evaluation of the specificity of the primers not included in Table 5 and 7 (local database). (PDF) [file pone.0096197.s012.pdf]

Table S6. *in silico* evaluation (percent matched 16S rRNA gene sequences in the target taxon) of the specificity of the primers not included in Table 5 and 7. Local database sequences were used for the evaluation.

Taxa	No. of sequences used for	Group specific primer				Archaeal universal primer			
		Cren457R	Cren499	Cren518R	CREN569	D33	A344F	Kb366F	W034
Crenarchaeota	872	42.5		86.5	10.6		9.4	2.9	
Euryarchaeota	6,072						17.0	1.8	2.6
Korarchaeota	88	1.1[a]							
Nanoarchaeota	3								
Thaumarchaeota	1,549			95.9	97.2				
FSCG	17			82.4	70.6				
HWCG-III	25			96.0	92.0				
MG-I	1,100			95.7	97.7				
RC	7			100	100				
SAGMCG-I	40			97.5	100				
SCG	355			96.6	96.9				
UT-I	2			100	100				
UT-II	1			100	100				
UT-III	2			100	100				
DSAG	326						0.3	0.3	
THSCG	50	2.0		92.0			≈0		
MCG	483			88.0	19.0		2.5	26.9	
UG	12								
Unclassified *Archaea*	272			42.3	40.8		22.8	1.8	1.1
Domain *Bacteria*[b]	667,899								

[a] Coverage values of more than 90% for target taxon are in bold, and tolerance values of more than 1% to non-target taxon are under-lined.

[b] Estimated using RDP's ProbeMatch.
