# Supplementary material for: Novel PCR Primers for the Archaeal Phylum Thaumarchaeota Designed Based on the Comparative Analysis of 16S rRNA Gene Sequences
Source: PLoS One. 2014 May 7;9(5):e96197. doi: 10.1371/journal.pone.0096197 (PMC4013054; doi:10.1371/journal.pone.0096197)
Supplement: Table S7 — In silico evaluation of the specificity of the primers not included in Table 5 and 7 (RDP database). (PDF) [file pone.0096197.s013.pdf]

Table S7. *in silico* evaluation (percent matched 16S rRNA gene sequences in the target taxon) of the specificity of the primers not included in Table 5 and 7. RDP database sequences were used for the evaluation because of the out-of-range (<300 or >1200) nucleotide positions of the primers.

Primer	Taxa						
	Crenarchaeota	Unclassified *Thermoprotei*	*Euryarchaeota*	*Korarchaeota*	*Nanoarchaeota*	Unclassified *Archaea*	*Bacteria*
Cren28F	5.7 (9[a]/157[b])	8.3(4/48)				12.5 (6/48)	
Cren7F	26.1(41/157)	20.8(10/48)				6.3 (3/48)	
89Fb	57.0(1,405/2,468)	74.5(1,273/1,709)	20.3 (1,005/4,939)	2.9 (1/35)		53.1 (492/926)	≈0
89F	11.0(273/2,482)	15.9(273/1,722)				37.2 (346/931)	
Cren113a	12.1(302/2,487)	17.2(297/1,726)	≈0				
Cren113	12.2(303/2487)	17.3(298/1,726)					
G-IV-1F	≈0	≈0					
Cren1209	0.7(6/825)	2.2(1/46)	≈0				
D30	7.6 (12/157)		51.1 (450/880)			4.2 (2/48)	
21F	27.4 (43/157)	20.8 (10/48)	65.1 (573/880)			6.3 (3/48)	
A3Fa	51.6 (81/157)	39.6 (19/48)	68.2 (600/880)			18.8 (9/48)	
A3Fb			83.9 (738/880)			4.2 (2/48)	
23F	72.0 (113/157)	56.3 (27/48)	80.7 (710/880)			33.3 (16/48)	
A25F	75.2 (118/157)	58.3 (28/148)	80.9 (712/880)			33.3 (16/48)	
Arch69F							
A109F	44.8 (1,106/2,468)	57.2 (977/1,709)	53.8 (2,655/4,939)	2.9 (1/35)		18.2 (169/929)	≈0
UA1204R	88.2 (2,308/2,618)	85.0 (1,563/1,839)	83.3 (5,172/6,209)	96.6 (85/88)	33.3 (1/3)	82.8 (784/947)	
EKb1242R	6.6 (173/2,618)	0.9 (17/1,839)	61.9 (3,842/6,209)			0.5 (5/947)	
Arch1381R	85.1 (1,376/1,617)	86.5 (1,088/1,258)	87.8 (3,948/4,496)			80.4 (483/601)	
arc1492r	59.1 (78/132)	14.6 (6/41)	81.7 (743/909)			18.7 (4/22)	70.0[c] (38,787/55,385)

[a] The number of matched sequences.

[b] The number of RDP sequences used for evaluation.

[c] Coverage values of more than 90% for target taxon are in bold, and tolerance values of more than 1% to non-target taxon are under-lined.
